# Interplay between high‐energy quenching and state transitions in *Chlamydomonas reinhardtii*: a single‐cell approach

**DOI:** 10.1111/nph.71001

**Published:** 2026-03-22

**Authors:** Aliénor Lahlou, Marcelo Orlando, Sandrine Bujaldon, William Gaultier, Eliora Israelievitch, Peter Hanappe, Thomas Le Saux, Ludovic Jullien, David Colliaux, Benjamin Bailleul

**Affiliations:** ^1^ CPCV, Department of Chemistry, École Normale Supérieure PSL University, Sorbonne University, CNRS Paris 75005 France; ^2^ Paris Research Sony Computer Science Laboratories Paris 75005 France; ^3^ UMR7141: Photobiology and Physiology of Plastids and Microalgae, Institut de Biologie Physico‐Chimique Sorbonne University, CNRS Paris 75005 France; ^4^ UMR7144: Adaptation and Diversity in the Marine Environment (ECOMAP), Station Biologique de Roscoff Sorbonne University, CNRS Roscoff 29680 France

**Keywords:** *Chlamydomonas reinhardtii*, high‐energy quenching, light stress response, machine learning, nonphotochemical quenching, photoinhibition, single‐cell photosynthesis, state‐transition

## Abstract

- Studying cell‐to‐cell heterogeneity is essential to understand how unicellular organisms respond to stresses. We introduce a single‐cell analysis framework that enables the study of intercellular heterogeneity of photosynthetic traits, particularly their interactions within individual cells that have identical genotypes, cellular contexts and histories.
- Our approach combines single‐cell imaging of Chl*a* fluorescence with machine learning, and we study light stress responses in *Chlamydomonas reinhardtii* as a proof of concept.
- This framework allows us to score the extent of high‐light responses such as state transitions (qT) and high‐energy quenching (qE). We reveal significant cell‐to‐cell heterogeneity and a strong correlation between qT and qE, undetectable in bulk measurements.
- This study highlights the value of single‐cell phenotypic analysis for investigating light stress responses in unicellular organisms. We detail the key aspects that come into play to generalize the method to other complex stress responses involving multiple traits.

## Introduction

It is crucial to study the correlations among traits in biological organisms as they may reveal important trade‐offs, which arose through evolution between various biological functions. These traits covariations may be difficult to study at the population level because of various factors that are difficult to control such as changes in the physiological state of the organisms. Single‐cell studies offer significant advantages for understanding the interplay between co‐occurring traits in a constant genetic background. When trait dispersion is sufficiently high, it provides a continuum of natural trait variations in synchronized isogenic cells in the same environment, a situation that is impossible to achieve when comparing two populations (Damodaran *et al*., 2015).

With regard to single‐cell tools, there has been a surge in the development of single‐cell sequencing technologies probing ‘omics’ data from genomics, proteomics, metabolomics, lipidomics and glycomics (Miao *et al*., 2021), all of them requiring cell lysing. A new challenge is to integrate them with noninvasive physiological measurements and further connect changes in gene expression and proteins, sugar and fat synthesis with physiological characteristics. For this purpose, being able to quantify a physiological process using a noninvasive and specific biological observable is highly desirable.

Chl*a* fluorescence (ChlF) emitted by photosystem II (PSII) is common to all photosynthetic organisms. It is the preferred observable for noninvasive studies of photosynthesis as it is highly sensitive and benefits from a century‐long history of research (Krause & Weis, 1984; Papageorgiou & Govindjee, 2004). After light absorption by the PSII light‐harvesting system, excited Chl can relax through three main de‐excitation pathways: photochemistry that initiates photosynthetic electron transfer, heat dissipation or emission of fluorescence – that can be measured optically (Grossmann & Wollman, 2023). Their relative efficiencies are determined by the values of the respective rate constants associated with each process. While the rate constant governing fluorescence emission remains invariant, the rate constants of the other processes can be modulated by biological factors. Photochemistry is modulated by the saturation level of the photosynthetic chain, while heat dissipation can be influenced by the regulated production of nonphotochemical quenchers in the light‐harvesting antenna. Because of such kinetic competition, variations of ChlF can provide valuable information on changes in photochemistry or heat dissipation and are used as a diagnostic tool to assess the overall status of photosynthesis (Krause & Weis, 1984; Papageorgiou & Govindjee, 2004).

Single‐cell photosynthetic responses are also instrumental to improve our basic understanding of photosynthesis. Most of the existing knowledge on photosynthesis has been derived from measurements at the organism or tissue level for plants, and bulk measurements for unicellular organisms (Cole *et al*., 2021). Those studies have revealed major insights into the biology of photosynthesis, yet they fail to account for the cell‐to‐cell heterogeneity, which is observed even within monoclonal populations (Brehm‐Stecher & Johnson, 2004; Lidstrom & Konopka, 2010). Accessing the biophysical heterogeneity at the single‐cell level reveals a richer set of information since the individual responses are not smoothed out by averaging (Küpper *et al*., 2000; Snel & Dassen, 2000; Šetlíková *et al*., 2005; Gachon *et al*., 2006; Trampe *et al*., 2011; Mohr *et al*., 2013; Damodaran *et al*., 2015; Konert *et al*., 2019; Westerwalbesloh *et al*., 2019; Behrendt *et al*., 2020; Andersson *et al*., 2021; Herdean *et al*., 2022; Széles *et al*., 2022).

In this work, we selected the response of photosynthetic organisms to high light (HL) stress as a case study. In natural environments, the intensity and wavelength distribution of sunlight fluctuates significantly over time scales ranging from seconds to months (Pearcy, 1990; Murchie *et al*., 2018). Several processes mitigate the risk of reactive oxygen species production and PSII photodamage under high light stress by regulating the photon absorption capacity of the photosystems and/or the efficiency of heat dissipation. These processes induce fluorescence changes and are referred to as nonphotochemical quenching (NPQ) (Minagawa & Tokutsu, 2015; Ruban, 2016). We chose to perform the analysis of those processes on the model unicellular green alga *Chlamydomonas reinhardtii* (Grossmann & Wollman, 2023) because this organism allows for easy isolation and manipulation of elementary processes involved in high light response. Here, we focus on two NPQ components and their associated biological processes (Goss & Lepetit, 2015):

- High‐energy quenching (qE), involving the rapidly reversible generation of a nonphotochemical quencher in the light‐harvesting antenna of PSII. In *C. reinhardtii*, qE occurs and relaxes within seconds and is regulated by conformational changes in the Light Harvesting Complexes Stress Related 1 and 3 (LHCSR1 and LHCSR3) induced by low lumenal pH.
- State transitions (qT), involving the reversible movement of a fraction of the light‐harvesting complexes between the photosystems. In state I, when all mobile Light Harvesting Complexes II (LHCIIs) are bound to PSII, the absorption cross section is large, resulting in high fluorescence levels. In state II, fluorescence decreases when LHCII detaches from PSII. It occurs and relaxes within minutes, regulated by LHCII phosphorylation via a kinase/phosphatase system (Wollman, 2001; Bergner *et al*., 2015; Cariti *et al*., 2020; Goldschmidt‐Clermont, 2023). Although qT is not a form of non‐photochemical quenching *per se*, it changes the absorption cross section of PSII with resulting variations of ChlF emission (Wollman, 2001; Goldschmidt‐Clermont, 2023).

We also took into account the contribution of slower NPQ components, in particular photoinhibition‐related quenching (qI), involving photodamaged PSII with a mechanism not yet fully understood (Nawrocki *et al*., 2021).

Rather than aiming to resolve the full biological mechanisms behind NPQ regulation, we use the well‐characterized responses in *Chlamydomonas* as a testbed to demonstrate how single‐cell variability can be used to explore interactions between traits. Population‐level studies typically use reverse genetics (or forward genetic screens) to create knockout mutants of one trait and examine the impact on other traits. However, mutagenesis can lead to secondary mutations and physiological changes and often fails to capture the complexity of trait interaction, such as nonlinearity or threshold effects. This study serves as a proof of concept for leveraging the often underestimated benefits of single‐cell analyses over population‐level studies by exploiting cell‐to‐cell heterogeneity to gain insights into the interplay between traits.

Our study aimed to answer the following questions: *What are the cell‐to‐cell variations in the extent of a given NPQ component when it is the only active*, *or between NPQ components when more than one is active? Can we benefit from cell‐to‐cell variations to explore the interaction between NPQ components?*

Established metrics have been empirically developed to quantify NPQ components from ChlF traces, but they often rely on basic mathematical treatments of specific values such as timestamps, inflection points or local extrema (Papageorgiou & Govindjee, 2004; Nilkens *et al*., 2010; Allorent *et al*., 2013; Tietz *et al*., 2017; Ruiz‐Sola & Petroutsos, 2018) or more recently on machine learning (Ramakers *et al*., 2025). To better separate contributions from concomitant processes, complementary observables such as fluorescence lifetime or multispectral acquisition can be introduced (Steen *et al*., 2022; Harris *et al*., 2024) but these strategies come at the cost of complexifying instruments and protocols. In this report, we present a computational method to analyze complex ChlF traces acquired with a CMOS camera and separate the contributions of co‐occurring processes. We make the *a priori* strong hypothesis that the behavior of a population (expressing multiple processes) can be satisfactorily accounted for by the referential of populations of a training dataset (expressing one elementary process). By selecting appropriate mutants, choosing specific growth conditions, or using customized treatments, we can build a dataset of reference populations, each exhibiting at most one NPQ component (qE, qT, qI or none). We trained an algorithm borrowed from the domain of image compression (Mairal *et al*., 2009) to extract the key ChlF patterns representing elementary kinetics. Then, we introduced an algorithm designed for class‐separability problems (Fisher, 1936) to project the ChlF traces into a three‐dimensional space where each axis represents an NPQ component. This transformation is then applied to wild‐type (WT) strains to score the three types of NPQ components (qE, qT, qI). This approach, based on well‐characterized responses to light stress, provides clear and unbiased scores for NPQ components in the WT strain. In our case study, we quantify the NPQ components in synchronized isogenic cells to investigate the cell‐to‐cell heterogeneity of qE and qT separately. Then, we exploit the observed cell‐to‐cell heterogeneity of qE and qT when they are co‐expressed to study the interplay between the underlying biological processes. While this study focuses on qE and qT in *Chlamydomonas*, the framework could be broadly applicable to other species and other types of stress responses involving multiple traits, provided that an appropriate training dataset can be generated.

## Materials and Methods

### Experimental design: optical calibration

#### Microscope setup

**Fig. 1:**
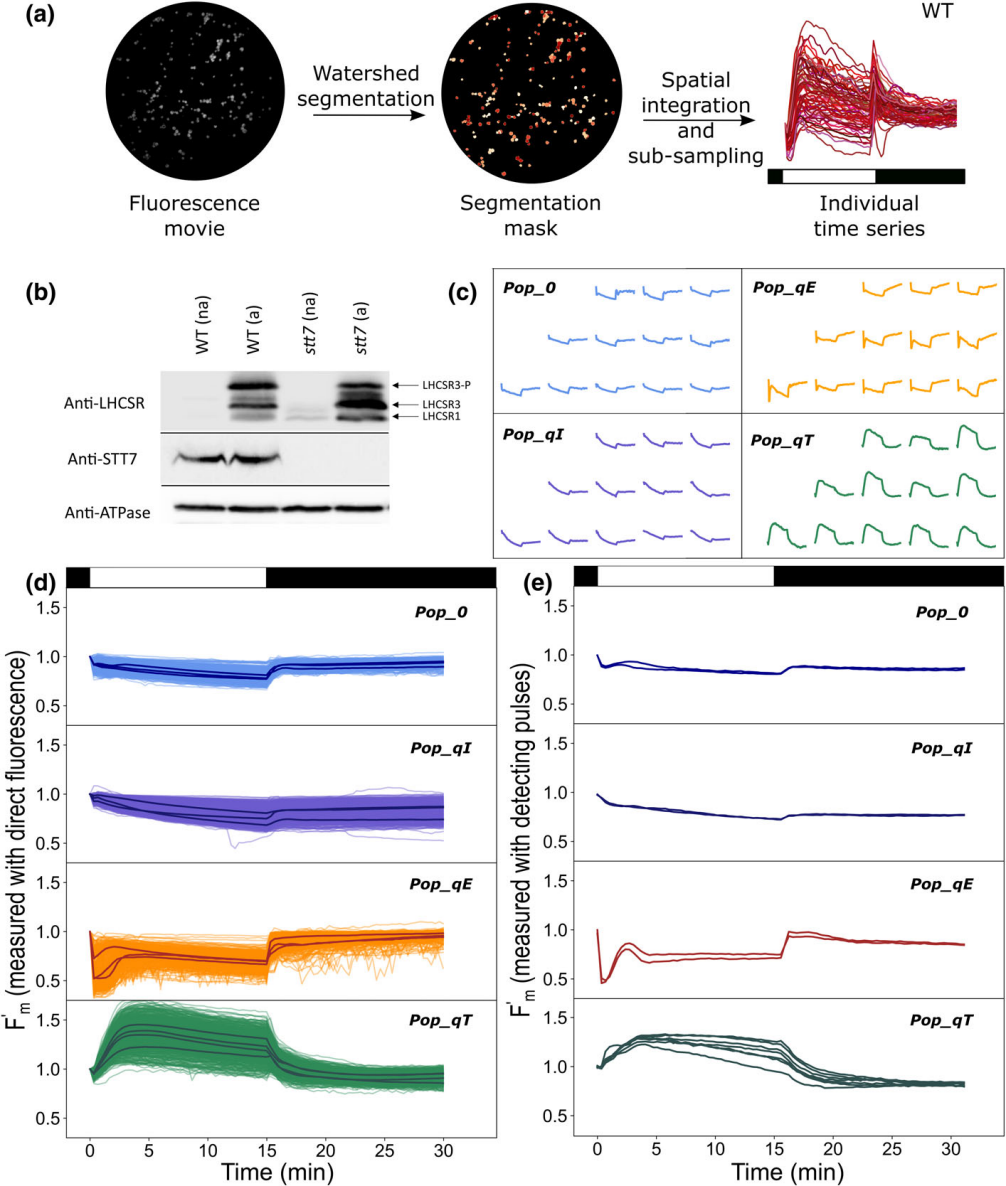
Acquisition of the reference dataset representing elementary *F*_m_′ traces for each nonphotochemical quenching (NPQ) component. (a) Extraction of the *F*_m_′ traces from single cells of the wild‐type (WT) imaged with the fluorescence microscope. Sample displaying qE and qT: population of the high light (HL)‐treated WT strain *wt4a* ^*−*^. The reference light protocol (15‐min HL–15‐min dark) is displayed as a black and white bar. (b) Immuno‐quantification of Light Harvesting Complexes Stress Related (LHCSR1, LHCSR3 and phosphorylated LHCSR3) and *serine/threonine protein kinase 7* (STT7) proteins, as well as AtpB (beta‐ATP synthase subunit) as a loading control. *Wt4a* ^−^ and STT7 knock‐out mutant (*stt7‐1*) were sampled as described in Methods, in low light (na, not activated) and after HL treatment (a, activated). (c) Randomly sampled maximal fluorescence yield (*F*_m_′) traces of the four populations used to build the reference dataset. Samples: populations of *Pop‐0* (untreated *stt7‐1* mutant, 3^rd^ repeat), *Pop‐qI* (untreated *stt7‐1* mutant, 1^st^ repeat), *Pop‐qE* (HL‐treated *stt7‐1* mutant, 4^th^ repeat) and (untreated *wt4a* ^*−*^, 4^th^ repeat); (d) *F*_m_′ single‐cell traces of the four populations used to build the reference dataset. The panels represent the superimposed single‐cell traces of > 400 cells from three independent biological replicates for *stt7‐1* and 4 for *wt4a* ^*−*^ (Table 1) and the average trace is shown as darker lines. (e) Validation of the microscope setup. *F*_m_′ traces measured on the same four populations as in (d) using a reference macro fluorescence imaging setup (see the Materials and Methods section) on two independent biological replicates for *stt7* *‐1* (5 for the *wt4a* ^*−*^). In (d, e), the *F*_m_′ data are normalized to the initial *F*_m_, in the dark‐adapted cell before the start of the actinic light period. The illumination protocol (HL: white boxes; dark: black boxes) are indicated on the top of the (a, d, e) panels.

We developed a fully automated Python‐based microscope capable of applying versatile illumination protocols to photosynthetic organisms (leaves or microalgae – see Supporting Information Fig. S1; Table S1). An Arduino board controls the 470 ± 10 nm and 405 ± 7 nm LEDs at the millisecond resolution. The blue LED is used for the high actinic light, the high‐light (HL) treatment and the relaxation, and the violet LED is used for the saturating pulses (SPs). We chose these wavelengths because it is possible to calibrate precisely their intensity with an actinometer‐based protocol (Lahlou *et al*., 2023). A Python algorithm segments with a watershed algorithm each cell from the first frame of the movie (Fig. 1a). Each cell mask is used to extract the mean fluorescence of the corresponding alga over the time frames and create a database of fluorescence traces for statistical analysis. See Supporting Information, for light calibration and more details (Figs S1–S3; Table S1).

#### Macro setup

Fluorescence emissions were measured using a Speedzen fluorescence imaging setup (JBeamBio, France) at room temperature with actinic light at 620 nm (740 μmol(photons)·m^−2^·s^−1^), SPs at 620 nm (5800 μmol(photons)·m^−2^·s^−1^, 250 ms), and weak blue ‘measuring’ light pulses (470 nm) to measure the fluorescence yield.

### Experimental design: biological samples

#### Strains

*Chlamydomonas reinhardtii* D. A. Dangeard (G.M. Smith) CC‐124 and *wt4a* ^*−*^ (CC‐4603) WT strains are derived from strain 137c, and the *npq4* mutant strain (CC‐4615, only presented in Fig. S4) were obtained from the Chlamydomonas Resource Center. *stt7‐1 #a6* mutant was from the ChlamyStation collection (http://chlamystation.free.fr/) and was obtained by crossing *stt7‐1* mutant from (Depège *et al*., 2003) with a WT (Depège *et al*., 2003; Bergner *et al*., 2015; Bujaldon, 2023). The strains were synchronized in a 12 : 12 light cycle (50 μmol(photons)·m^−2^·s^−1^, 23*°*C) and grown in mixotrophic conditions (Tris‐Acetate‐Phosphate medium (Gorman & Levine, 1965)). The synchronized strains grown on Petri dishes were inoculated in liquid medium under agitation for 2 d. The culture was diluted with fresh Tris‐Acetate‐Phosphate. The experiments were always performed in the exponential phase, and the whole illumination protocol started *c*. 2 h after the beginning of the light phase of the photoperiod.

#### Pharmacological inhibitors

Lincomycin (Sigma‐Aldrich) was diluted in water and used at a final concentration of 1 mM (see Fig. S5).

#### Sample preparation for microscope experiments

In all experiments, samples are collected from cultures in exponential phase (from 5 *×* 10^5^ to 5 *×* 10^6^ cells·ml^−1^), centrifuged (960 ***g*** for 8 min), and transferred to Tris‐minimal (MIN) medium at *c*. 10^7^ cells ml^−1^. Such a centrifugation step does not affect photosynthetic physiology – provided that cells are allowed to rest for at least 15 min after centrifugation, which is the case here – and is required to allow the measurement of > 100 cells in the field of view. The algae were deposited on agarose pads for observations based on protocol from (Chouket *et al*., 2022). A 1% suspension of agarose in MIN medium was heated to dissolve the agarose. The agarose pads were prepared by dropping 175 μl of melted agarose onto a glass slide holding a spacer (AB‐0578; ThermoFisher, Waltham, MA, USA). To make its surface flat, the pad was covered with a thick microscope glass slide and left for 5 min at 4*°*C for hardening. The cover slide was removed and the preparation was left for 10 min to allow thermal adaptation and evaporation of excess humidity. About 5 μl of algae suspension was dropped onto the agarose and left uncovered for 10 min at room temperature to remove excess water. Finally, a coverslip was gently placed over the agarose pad for observation. A side opening in the spacer ensured gas exchanges during the experiments (Fig. S6). We checked that cells grew on those agarose pads (Fig. S6), keeping their synchronicity (Fig. S7).

#### Sample preparation for macro setup experiments

Cells are centrifuged and resuspended in MIN (same protocol as above) at a final concentration of 2 *×* 10^7^ cells·ml^−1^. After at least 30 min of relaxation under the growth conditions, they are either used directly, or high‐light pre‐treated as explained below. For observations under the SpeedZen, the cell suspensions are placed in a metal plate inside open cavities of 85 μl.

#### Monoclonal cultures

The synchronized monoculture of *stt7‐1* and *wt4a* ^***−***^ were obtained by the streak plate protocol (Katz, 2008). The populations were subcloned once. The mother *wt4a* ^***−***^ population had not been subcloned for more than a year, while the mother *stt7‐1* population had been obtained by crossing less than 1 yr before.

#### Analysis of polypeptides

Cells were grown, harvested and conditioned according to the conditions described for macro setup experiments. The population of *wt4a* ^*−*^ and *stt7‐1* was harvested (2 × 10^7^ cells) in low light (na) and after HL treatment of 4 h (a). Gel extraction was performed following a classical protocol (Piccioni *et al*., 1981). Immunodetection was performed using antibodies against LHCSR (Bonente *et al*., 2011), STT7 (Bergner *et al*., 2015) and AtpB (beta‐ATP synthase subunit) (Atteia *et al*., 1992) (See Supporting Information, for more details).

### Experimental design: light protocols

#### Reference protocol

The algae are exposed to 15 min of 400 μmol(photons)·m^−2^ s^−1^, 470 *±* 10 nm, and 15 min of dark. Throughout the whole experiment, SPs are shone every 20 s (1400 μmol(photons)·m^−2^·s^−1^, 405 *±* 7 nm, 200 ms). The whole fluorescence is collected with a camera, algae were selected from the video using watershed segmentation (see Supporting Information; Fig. S8) and only the response specific to SPs, *F*_m_′, is analyzed for each segmented cell. The actinic light is turned off when the SP is applied, and we verified that SPs alone did not affect the *F*_m_′ traces (the light protocols are illustrated in detail in Figs S9–S11; Table S2). The four repeats of the reference protocol and the HL treatment are performed under the microscope without displacement of the sample. The last two repeats are considered qI‐free because the *F*_m_ level at the beginning of the exposure is very close to the *F*_m_′ level at the end of the exposure (Fig. S11).

#### HL treatment

The algae deposited on a pad are exposed to 400 μmol(photons)·m^−2^·s^−1^, 470 *±* 10 nm for 1 h 20 min–4 h under the microscope. The HL treatment is followed by 45 min of 40 μmol(photons)·m^−2^·s^−1^, 470 *±* 10 nm. For macro setup experiments, the algae suspensions are subjected to 550 μmol(photons)·m^−2^·s^−1^ of white light for 4 h under strong agitation. The HL treatment is followed by 45 min of relaxation under low light (15 μmol(photons)·m^−2^·s^−1^, white LED panel).

### Data analysis

Before the machine learning protocol, the outliers were removed from the training dataset. The algae with a surface smaller than 5 pixels were discarded. Then, a KD‐Tree (Bentley, 1975) was built to represent the Euclidean distance relationships between the inputs. We manually set a threshold *D* = 0.01 to remove the points with all neighbors further than *D*. In total, 66 *F*_m_′ traces out of 2302 were removed from the training dataset.

#### Dictionary learning

**Fig. 2:**
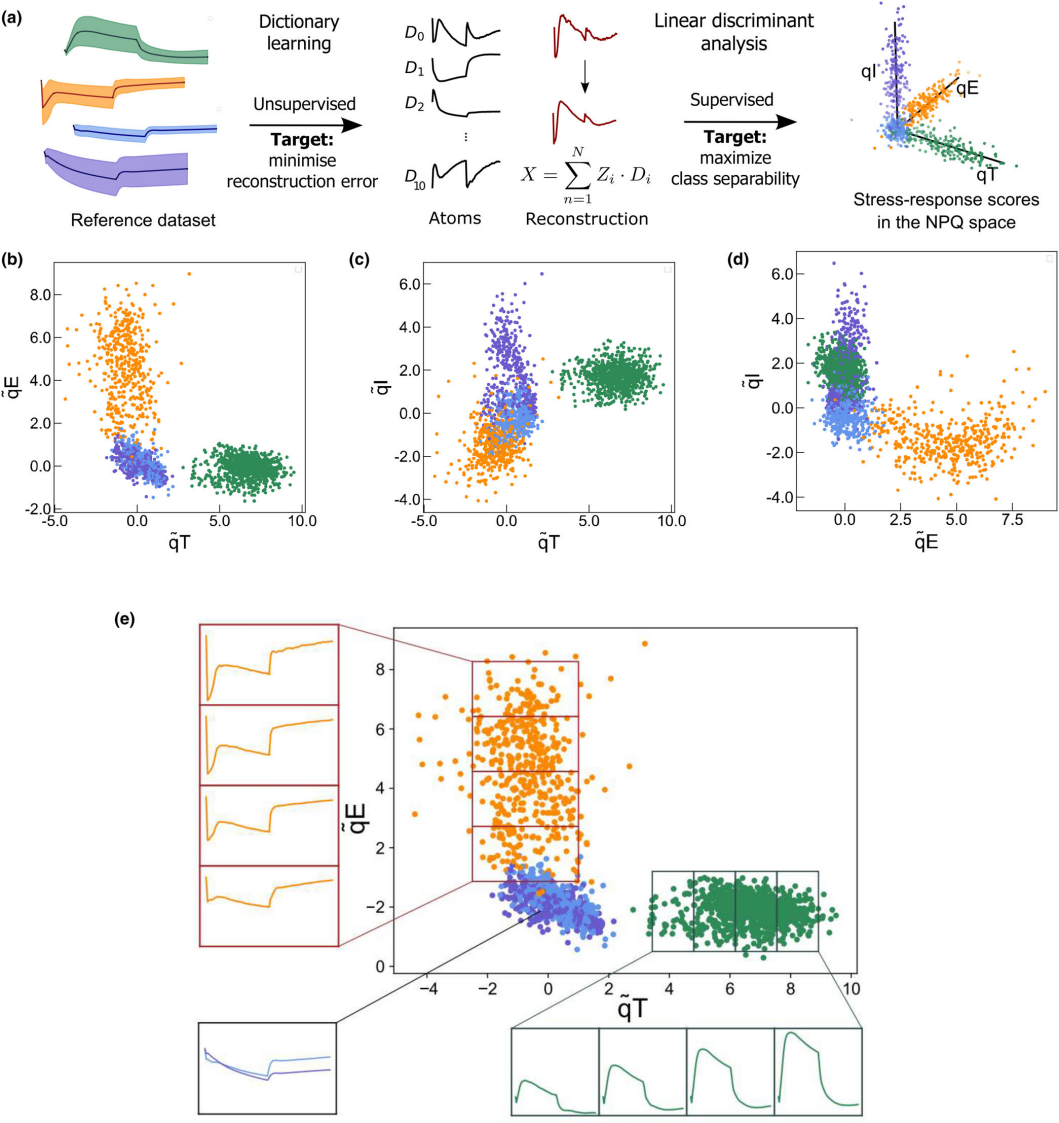
From the reference dataset to a base of elementary nonphotochemical quenching (NPQ) components in a 3D space (dataset: see Table 1). (a) Flow of the process. A subset of the reference dataset traces is first used to perform unsupervised training of an algorithm of dictionary learning, which learns to reconstruct the traces with a minimal set of elementary traces (atoms). The resulting reconstructed traces are then combined with the labels of the four training populations to perform a linear discriminant analysis. It results in a vector in a 3D space, where each axis corresponds to a population of the reference dataset expressing an elementary stress. The axis is named according to this corresponding population: q̃T, q̃E or q̃I; (b–d) 2D projections of the reference dataset onto the 3D NPQ space after performing linear discriminant analysis on the atom code (Dimension 10) from applying dictionary learning. (b) (q̃T, q̃E), (c) (q̃T, q̃I), (d) (q̃E, q̃I). Orange: *Pop‐qE*, green: *Pop‐qT*, blue: *Pop‐0*, purple: *Pop‐qI*: (e) Qualitative analysis of the evolution of the *F*_m_′ traces along the q̃E and q̃T axis. The orange population corresponds to the population *Pop‐qE* (HL‐treated *stt7‐1*, fourth repeat). The span of (98% of) the population along the q̃E axis is split into four equal parts, and the average *F*_m_′ trace within each quarter are computed using the *F*_m_′ traces (normalized to the first point) corresponding to the data points falling inside the quarter. The same is performed for the *Pop‐qT* population (green point cloud, untreated *wt4a* ^*−*^ fourth repeat) along the q̃T axis. For comparison, the average of the *F*_m_′ traces (normalized to the first point) of the *Pop‐qI* (purple) and *Pop‐0* (blue) populations are also plotted (untreated *stt7‐1*, first and third repeat resp.). For all plots, the span of the y‐axis is equal to 0.7, with the first point falling at Position 1.

Dictionary learning (Figs 2a, S12, S13; Mairal *et al*., 2009) is used to decompose any complex series of data into prelearned arbitrary waveforms that capture its most important features. The training phase is unsupervised: It exploits all the *F*_m_′ traces from the dataset without any label. It learns to identify characteristic patterns in the ChlF traces that are stored in a dictionary of basic waveforms (called atoms). Its metric is the accuracy of reconstruction of a ChlF trace as a linear combination of the atoms. We used the sparse coding method to decompose the data into a linear combination of atoms from a dictionary (Mairal *et al*., 2009) implemented in *scikit‐learn* (Pedregosa *et al*., 2011). The coefficients weighting the atoms form a feature vector. The dimension of this feature vector is lower than the dimension of the original *F*_m_′ trace (91) and is equal to *N*_D_, the number of atoms in the dictionary. To build the dictionary, the randomly sampled balanced dataset containing *n*_samples_ *F*_m_′ traces from *Pop‐qE*, *Pop‐qT*, *Pop‐qI* and *Pop‐0* was used as a training dataset and fitted with least angle regression following (Mairal *et al*., 2009). The metric optimized is the reconstruction fidelity with a penalty on the sparsity level, by minimizing the following function:

(Eqn 1) $$\min_{D,X}\frac{1}{2}\;{\left\|Y-\mathrm{DX}\right\|}_{2}^{2}+\lambda {\left\|X\right\|}_{1}$$

where **Y** is the input data matrix of dimension 91*× n*_samples_, **D** is the dictionary matrix with *N*_D_ atoms of dimension 91 (normal matrix), **X** is the coefficient matrix of dimension $N_{D}\times n_{\text{samples}}$, ${\left\|Y-\mathrm{DX}\right\|}_{2}^{2}$ is the squared Euclidean norm of the reconstruction error, ||**X**||_1_ is the L1 norm (sum of absolute values) of the coefficients, *λ* is the regularization parameter that controls the sparsity level.

We explored several parameters detailed (Fig. S12) including the normalization of the *F*_m_′ traces before the training. For the analysis described in the main text, we selected *N*_D_ = 10, *n*_samples_ = 300, *λ* = 10^*−*6^ and normalized by the sum of the *F*_m_′ to obtain a probability distribution.

#### Linear discriminant analysis

The dimension reduction algorithm, multiclass linear discriminant analysis (Fisher, 1936) (LDA) finds the best linear combinations of input features that maximize the separability among differently labeled data. The metric optimized is derived from two matrices, *S*_B_ and *S*_W_, which represent respectively the separation between (B) the mean of the classes and the dispersion of the datapoints within (W) each class. We obtain a projection from a space of dimension 10 to a space of dimension *c*_*−*_ 1 where *c* is the number of classes (in our case *c* = 4, *Pop‐qE*, *Pop‐qT*, *Pop‐qI* and *Pop‐0*). The LDA is solved by singular value decomposition (scikit‐learn Python library) (Pedregosa *et al*., 2011). Once the projection **T** ∈ 10 *×* 3 is obtained, we operate a transformation to align the significant axis with the canonical space (*x*, *y*, *z*) for easier analysis. The first step is to identify the principal directions of each class in the dataset and associate it with an axis by collecting the corresponding eigenvector, using principal component analysis (Duda *et al*., 1973). They form a transfer matrix **R**, which allows changing the base of the space. Then, the origin is identified by using biological knowledge. The average of the point clouds representing *Pop‐qE*, *Pop‐qI* and *Pop‐0* is expected to show no qT as the strain *stt7‐1* is an serine/threonine protein kinase 7 (STT7) knockout mutant: It is the origin of the q̃T axis. The average of the point cloud *Pop‐qI* is expected to show no qE because it has only been exposed to growth light. It is set as the origin of the axis q̃E. The average of the point clouds *Pop‐qE*, *Pop‐qT* and *Pop‐0* is supposed to show no qI since the last *F*_m_′ equals the first *F*_m_ and is therefore used as the zero reference for the q̃I axis.

#### Evaluation of the cell‐to‐cell vs cell‐to‐population distances

**Fig. 3:**
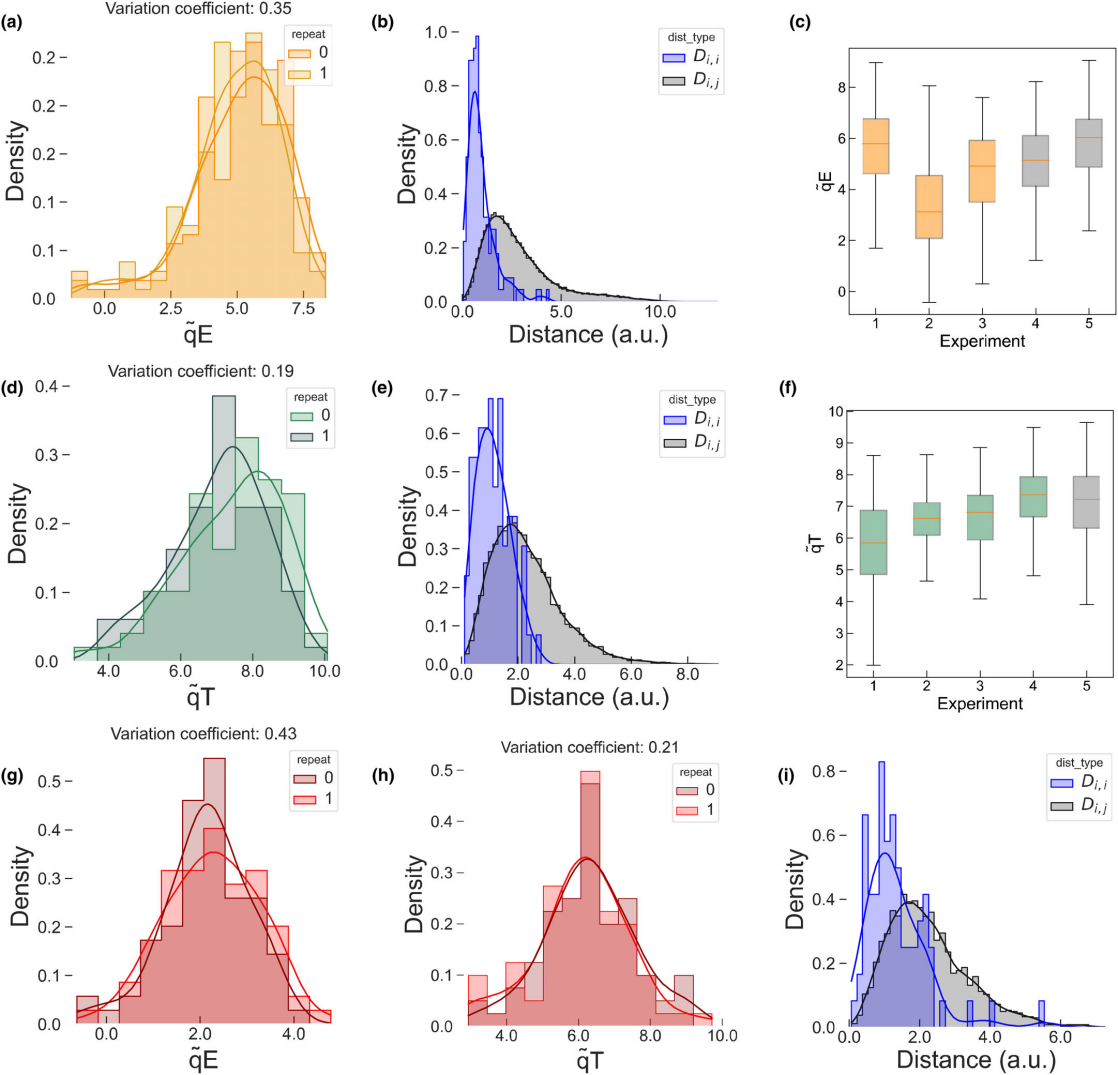
Intercellular variations in qE and qT in monoclonal synchronized populations. (a, d) Distribution of the q̃E (a, 176 algae) and q̃T (d, 77 algae) values of two consecutive repeats of the same experiment on isogenic and synchronized cells of the HL‐treated *stt7‐1* (a) and untreated wild‐type (WT) *wt4a* ^***−***^ (d) without qI. We evaluate the variation coefficient at 19% for qT, 35% for qE; (b, e) distributions of the cell‐to‐cell (*D*_*i*,*i*_ – blue, SD: 0.7 (b), 0.3 (e)) vs cell‐to‐population (*D*_*i*,*j*_ – black, SD: 1.8 (b), 1.5 (e)) distances in the 3D nonphotochemical quenching (NPQ) space retrieved from (b) two isogenic high light (HL)‐treated *stt7‐1* populations (2 consecutive repeats; 176 algae); (e) HL‐treated *wt4a* ^***−***^ population without qI (2 consecutive repeats; 77 algae); (c) dispersion of the q̃E values collected from the training dataset (orange – 1: 111 cells, 2: 145 cells, 3: 224 cells) and experiments with isogenic synchronized populations (gray – 4: 176 cells, 5: 231 cells); (f) dispersion of the q̃T values collected from the training dataset (green – 1: 177 cells, 2: 365 cells, 3 : 195 cells, 4: 208 cells) and experiments with isogenic synchronized populations (gray – 5: 77 cells); (g, h) distribution of the q̃E (g) and q̃T (h) values of two repeats of the same experiment on isogenic and synchronized cells of the HL‐treated *wt4a* ^***−***^ populations without qI. We evaluate the variation coefficient at 21% for qT, 43% for qE; (i) distributions of the cell‐to‐cell (*D*_*i*,*i*_ – blue, SD: 0.7) vs cell‐to‐population (*D*_*i*,*j*_ – black, SD: 1.3) distances in the 3D NPQ space retrieved from two isogenic *wt4a* ^***−***^ populations (2 consecutive repeats; 77 algae). See Supporting Information Table S3 for all statistical tests related to the figure. In (c, f) error bars represent the SE, the boxes show the interquartile range (IQR, middle 50% of the data), the central line indicates the median, whiskers extend to 1.5 times the IQR.

As the experiments are performed on the same sample with *N* algae, we can derive the couple positions in the dictionary learning NPQ space ((*x*_3,*i*_,*y*_3,*i*_,*z*_3,*i*_),(*x*_4,*i*_,*y*_4,*i*_,*z*_4,*i*_)), *i* ∈ [1,*N*] corresponding to algae *i* for the third and fourth repeats (Fig. 3b,e,i). We introduce the distance *D*_*i*,*j*_ as the distance of the point corresponding to algae *i* in the third repeat to the point corresponding to algae *j* in the fourth repeat:

(Eqn 2) $$D_{i,j}=\begin{array}{l} \sqrt{{\left(x_{4,j}-x_{3,i}\right)}^{2}+{\left(y_{4,j}-y_{3,i}\right)}^{2}+{\left(z_{4,j}-z_{3,i}\right)}^{2}}\;\text{for}\left(i,j\right)\\ \in \left[1,N\right] \end{array}$$

#### Grid representation of the *F*_m_ traces in the NPQ space

**Fig. 4:**
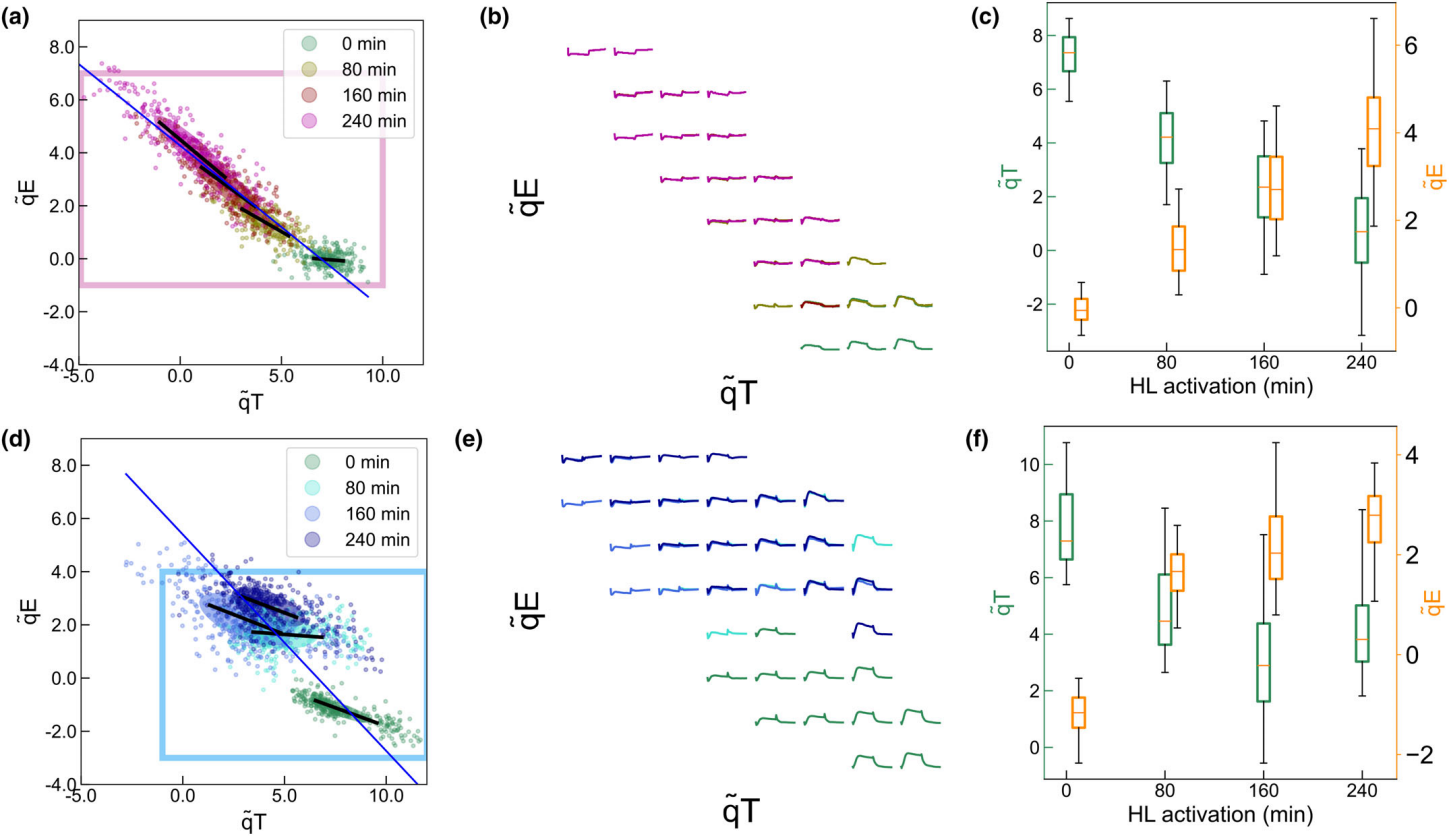
Evolution of the distribution of wild‐type (WT) nonisogenic populations throughout high‐light ‐treatment (HL activation). (a) WT *wt4a* ^***−***^ population taken from the growth light not HL‐treated and displaying minimal qI component (untreated *wt4a* ^***−***^ equivalent to *Pop‐qT*, 4^th^ repeat, 196 cells) exposed to three consecutive HL treatment protocols of 80 min to promote the expression of Light Harvesting Complexes Stress Related 3 (LHCSR3) protein and induce qE. The blue line indicates the direction of the linear fitting curve of the point cloud centroids while the black line indicates the linear curve fit of each point cloud separately; (b) grid projection of the *F*_m_′ traces from (a); (c) amplitude of the trait along the q̃T axis (left, green) and q̃E axis (right, orange) throughout the HL treatment of WT *wt4a* ^***−***^. (d) WT *cc124* population taken from the growth light not HL‐treated and displaying minimal qI component (4^th^ repeat, 335 cells) exposed to three consecutive HL treatment protocols of 80 min to promote the expression of LHCSR3 proteins and induce qE. The blue line indicates the direction of the linear fitting curve of the point cloud centroids while the black lines indicate the linear curve fit of each point cloud separately; (e) grid projection of the *F*_m_ *′* traces from (d); (f) amplitude of the trait along the q̃T axis (left, green) and q̃E axis (right, orange) throughout the HL treatment of WT *cc124*. In (b, d), the axis limits have been adjusted to the rectangles (red, a – blue, d) and a trace is represented in the grid only if more than three datapoints fall within the box. In (c, f) the boxes show the interquartile range (IQR, middle 50% of the data), the central line indicates the median, whiskers extend to 1.5 times the IQR. See Supporting Information Table S3 for statistical tests related to correlation at the population and single‐cell level in (a, d).

To build Fig. 4(b,e), we grouped the data points in an 8 *×* 8 grid and plotted the average *F*_m_′ trace if there were more than three data points in a grid element. The traces from different point clouds are overlaid with the corresponding colors indicated in Fig. 4(a,d).

## Results

### New instrument and protocol to probe high‐light responses in single cells

We developed a fully automated microscope capable of applying versatile illumination protocols to capture ChlF traces from microalgae immobilized on agarose (see the Materials and Methods section). Our light protocol consists in exposing the algae to high‐light and applying SPs every 20 s to probe the SP‐induced fluorescence (Goss & Lepetit, 2015). Since the number of photons exciting the system during the SP is constant, we use the SP‐induced fluorescence to track the evolution of the maximal fluorescence yield (*F*_m_ before illumination and *F*_m_′ during and after HL treatment, see the Materials and Methods section). Because the yield of photochemistry becomes negligible during SP, changes in the maximal fluorescence yield, *F*_m_′, reflect changes in the efficiency of the competing nonphotochemical processes (Goss & Lepetit, 2015). We applied an image segmentation algorithm to retrieve the ChlF temporal trace of each alga, called *F*_m_′ trace hereafter (Fig. S8). In Fig. 1(a), we display an example of individual *F*_m_′ traces of a WT population expressing qE and qT when exposed to our reference protocol, which consists in 15‐min exposure HL followed by 15 min of relaxation in the dark several times in a row. We chose the strain *wt4a* ^*−*^ in this work, otherwise specified.

### Building a reference dataset of elementary qE, qT and qI traces

We developed a machine learning‐based workflow to accommodate the variability in the kinetic properties of each NPQ component. Our approach assumes that the ChlF trace of a cell expressing multiple processes, such as in WT (Fig. 1a), can be modeled as a linear combination of elementary traces. Therefore, we leveraged extensive knowledge and resources on high‐light stress responses in *C. reinhardtii* to establish a training dataset that is strains and conditions in which particular NPQ traits are either present or absent (Allorent *et al*., 2013; Roach & Na, 2017; Steen *et al*., 2022). We defined three distinct populations – specific strains conditioned under appropriate settings – each expressing a single dominant NPQ component, with minimal expression of the others. These populations provided elementary traces representing a single NPQ component, capturing variability in both amplitudes and time constants. Combined with a fourth population lacking all NPQ components, these traces formed the training dataset (see the Materials and Methods section; Table 1, Fig. 1). We verified that *F*_m_′ traces were induced by the HL treatment and not by the SPs applied every 20 s (Fig. S10).

**Table 1:** Description of the dataset (strains, conditioning, and acquisition).

Name	No. of experiments	Total no. of algae	No. of replicates	Strain	HL treatment	Reference protocol repeat	qT	qE	qI
*Pop‐0*	3	433	3	*stt7‐1*	**∅**	3^rd^			
*Pop‐qI*	3	453	3	*stt7‐1*	**∅**	1^st^			**✓**
*Pop‐qE*	3	480	3	*stt7‐1*	**✓**	4^th^		**✓**	
*Pop‐qT*	4	936	4	*wt4a* ^*−*^	**∅**	4^th^	**✓**		

*Pop‐0*: untreated *stt7‐1* (third repeat of the reference protocol); *Pop‐qI*: *untreated stt7‐1* (first repeat); *Pop‐qE: high‐light‐treated* (*HL‐treated*) *stt7‐1* (fourth repeat); *Pop‐qT: untreated wt4a* ^*−*^ (fourth repeat). Shaded cells: NPQ component absent, checkmark: NPQ component present/HL treatment applied, ∅: no HL treatment.

For our experiments, we used cells grown under low light in acetate‐containing medium to create qE‐free conditions (‘untreated’ hereafter), as these conditions suppress LHCSR gene expression (see western blot in Fig. 1b and (Peers *et al*., 2009; Allorent *et al*., 2013; Ruiz‐Sola & Petroutsos, 2018; Ruiz‐Sola *et al*., 2023)). By contrast, to induce qE, we subjected cells to a 1 h 20 min–4 h HL treatment under the microscope after transferring them to acetate‐free medium (see the Materials and Methods section), which triggers LHCSR production (‘treated’ hereafter; Fig. 1b and Peers *et al*., 2009; Allorent *et al*., 2013; Ruiz‐Sola & Petroutsos, 2018). To study conditions without qT, we used the kinase STT7 knockout mutant, which remains blocked in state I. We use the WT strain (*wt4a* ^*−*^) to study conditions with qT and the STT7 knockout mutant (*stt7‐1* #a6 obtained from multiple backrosses of *stt7‐1* mutant strain (Depège *et al*., 2003) with wild‐type strain (Bujaldon 2023)), to study conditions without qT (see STT7 quantification by western blots in Fig. 1b).

Based on this, the untreated STT7 knockout mutant was expected to lack both qE and qT. However, we observed a significant decrease of *F*_m_′ in this population during the 15‐min HL period, which did not relax during the subsequent dark period (Fig. S11). Several slowly relaxing NPQ components have been described, including qI quenching (Nawrocki *et al*., 2021), xanthophyll cycle‐dependent qZ quenching (Nilkens *et al*., 2010; Kress & Jahns, 2017) and sustained qH quenching (Malnoë, 2018). However, key proteins involved in qH quenching are absent in the *C. reinhardtii* genome (Brooks, 2012; Malnoë, 2018). This slowly induced and slowly relaxing quenching was exacerbated by the chloroplastic translation inhibitor lincomycin (Fig. S5), consistent with qI quenching (Nawrocki *et al*., 2021). PSII photodamage and qI occur when excessive light disrupts the balance of ongoing degradation and repair processes of reaction centers, particularly the D1 protein encoded in the chloroplast genome (Tyystjärvi & Aro, 1996; Tyystjärvi, 2013). Therefore, we attribute the slowly reversible quenching observed in our dataset to qI, although a contribution from qZ cannot be strictly excluded (Nilkens *et al*., 2010). Manipulating qI is more challenging than manipulating other NPQ components, as there are no mutants providing qI‐free ChlF traces. Although the primary goal of this study was to investigate the heterogeneity and interaction of qE and qT, the inclusion of elementary traces for qI in the training dataset was essential for the machine learning‐based approach to work effectively. We found that repeated exposure to our reference protocol (15‐min HL–15‐min dark) progressively reduced the extent of qI, likely reaching a steady state in which PSII repair balanced PSII damage (Tyystjärvi, 2013). Therefore, for our training dataset, we used the third or fourth iteration of the reference protocol to create conditions without qI contribution and the first iteration to represent conditions with qI.

We used the above considerations to generate the required four populations expressing only qE, only qT, only qI or none of those (Table 1). In practice, we used the fourth repeat of our reference protocol on the untreated WT population to obtain elementary traces of qT (*Pop‐qT*), as it expresses the STT7 kinase but not LHCSR3 proteins. The fourth repeat of our reference protocol on the HL‐treated *stt7‐1* mutant provided elementary traces of qE (*Pop‐qE*) since this mutant then expresses LHCSR3 proteins but lacks STT7 kinase. In the untreated *stt7‐1* population, the first repeat was used for the elementary traces of qI (*Pop‐qI*), whereas the third repeat was used as the reference population displaying none of the NPQ components (*Pop‐0*). Indeed, qE slightly increased during the repeats of 15‐min illumination needed to reduce the contribution of qI and we came up with using the third repeat instead of the fourth as the best compromise (Figs 1b, S11b). This was probably due to the fact that the induction of LHCSR proteins by light is more sensitive in the *stt7‐1* strain (Bonente *et al*., 2011; Allorent *et al*., 2013).

Fig. 1(c) displays several randomly sampled traces for the four populations, demonstrating the diversity of the ChlF responses in the training dataset. The whole dataset is plotted in Fig. 1(d) for *Pop‐qE*, *Pop‐qT*, *Pop‐qI* and *Pop‐0*. These *F*_m_′ traces were collected from movies displaying 100–300 algae cells in at least three independent biological replicates (see the Materials and Methods section; Table 1; Fig. S6, for sample preparation details). The algae populations were synchronized but not isogenic (see the Materials and Methods section). Mean responses calculated from individual traces of each biological replicate are shown as a black line in Fig. 1(d). The data convincingly reproduce the classical trends of the NPQ components (Allorent *et al*., 2013; Roach & Na, 2017; Ruiz‐Sola & Petroutsos, 2018; Steen *et al*., 2022):

- *Pop‐qE* traces show a steep decay at the onset of light and rapid relaxation of the decline in *F*_m_′ at the end of light consistent with the contribution of qE. In most cells, qE reached a transitory maximum before partially relaxing during the HL period. Such transitory qE has been reported in *Chlamydomonas* (Bonente *et al*., 2011; Roach & Na, 2017; Steen *et al*., 2022) or in plants, in which the relaxation of qE in the light was attributed to the concomitant activation of carbon fixation in the Calvin–Benson–Bassham (CBB) cycle (Finazzi *et al*., 2004).
- *Pop‐qT* traces show a gradual increase of *F*_m_′ at the onset of light and a gradual relaxation during the dark period, both phases lasting a few minutes, indicating qT and the movement of LHCII from PSI (state II) to PSII (state I) in the HL period, and back to PSI in the dark. It might seem counter‐intuitive that LHCIIs switch from PSI to PSII under high‐light conditions. Indeed, PSII is already under excessive light pressure, and this should favor PQ pool reduction, STT7 activation and state II. However, this behavior has been documented previously (Allorent *et al*., 2013) and attributed to kinase inactivation by negative redox control by the ferredoxin/thioredoxin system (Rintamäki *et al*., 2000) or to steric hindrance preventing LHCII kinase phosphorylation due to HL‐induced conformational changes in PSII (Vink *et al*., 2004).
- *Pop‐qI* traces show a continuous decline of *F*_m_′ during the HL period, which does not fully recover during 15 min of darkness, characteristic of the contribution of qI, with an overall decay between the initial and final *F*_m_′.
- *Pop‐0* is not completely flat as would be expected if all NPQ components were absent. However, the slight decrease of *F*_m_′ at the light onset, representative of qE, and the slight decay of *F*_m_′ between the beginning and the end of the experiment, representative of qI, are both negligible compared to their equivalent in the *Pop‐qE* and *Pop‐qI*, respectively.

Moreover, these results compare well with the *F*_m_′ traces measured at the population level using a conventional imaging fluorometer (macro setup, see the Materials and Methods section) by following the same light and dark protocols, including the actinic and saturating pulse sequences (Fig. 1e) (Johnson *et al*., 2009; Allorent *et al*., 2013).

### Using the reference dataset to build a 3‐dimensional (qE, qT, qI) space

We used the reference dataset with a machine learning framework to derive a quantitative estimate of the three NPQ components from any ChlF trace. This process involves constructing a 3D space in which the three axes represent the elementary biological processes associated with the NPQ components qE, qT and qI, respectively (3D NPQ space hereafter). The framework performs two consecutive dimension reduction steps, each optimizing a different metric, to transform the *F*_m_′ traces into a biologically interpretable 3D vector. Once trained, the framework can be used to analyze new data: An input *F*_m_′ trace is first reduced to a vector that describes the linear combination of basic waveforms reconstructing the *F*_m_′ trace. This vector is then projected onto a 3D space with axes denoted q̃T, q̃E and q̃I (Fig. 2a), attributing a score to each NPQ component.

The first step of dimension reduction aims at learning a minimal set of ChlF waveforms from the training dataset to reconstruct generic input ChlF traces with minimal reconstruction error. This step is necessary to validate our working hypothesis stating that a trace presenting co‐occurring NPQ components can be interpreted as a combination of elementary traces associated with each NPQ component (see the Discussion section). We used a dictionary learning method (Mairal *et al*., 2009) and selected the hyperparameters to minimize the reconstruction error (see the Materials and Methods section; Figs S12, S13). Although the training set contains four populations, we selected an optimized dictionary that includes 10 atoms, based on a reconstruction error threshold (2 × 10^*−*4^; Fig. 2a). This larger dictionary allows for linear combinations of trends that better represent the variability present in the training data (see *F*_m_′ traces in Fig. 1c) and offers the flexibility needed to capture the dispersion of kinetic parameters within each NPQ component – something a dictionary limited to four representative atoms would not allow (Fig. S12C,D).

The second step is a supervised dimension reduction aimed at maximizing the separability of the annotated training populations. This step learns a matrix to project the output of the first step onto a 3D space using a method based on LDA (see the Materials and Methods section) (Fisher, 1936). LDA learns a projection matrix from the 10‐dimensional dictionary output space to a 3D space by identifying three hyperplanes that effectively separate the four training populations. This dimensionality constraint explains the need to select four classes to construct the reference dataset. Once the 3D projection was obtained, we defined the final 3D basis of the NPQ elementary components by assigning the *x*, *y* and *z* axes to the directions along which the training populations *Pop‐qE*, *Pop‐qT* and *Pop‐qI* are spread (principal components) and redefining the origin thanks to the population *Pop‐0*. Therefore, the point cloud of each training population extends along a separate axis and, since each population represents an elementary HL stress‐response, the axes were named q̃T, q̃E or q̃I according to the corresponding NPQ components (see the Materials and Methods section).

The 3D projection results of our machine learning framework applied to the training dataset are shown in Fig. 2(b–d) with each panel representing a 2D projection for clarity. The scatterplots of populations expressing the qT or qE component extend only along the q̃T or q̃E axis respectively; those not expressing the component overlap at the origin of the corresponding axis. In the plane (q̃T, q̃E), the populations *Pop‐qI* and *Pop‐0* overlap since they exhibit neither qE nor qT (Fig. 2b). The scatterplot of the population *Pop‐qI* along the q̃I axis is also consistent, even though it is less separated from the other populations compared to the other axis (Fig. S14).

The two consecutive steps applied to the training dataset allow the qualitative separation of point clouds corresponding to populations exhibiting distinct NPQ components. Beyond class separation, this representation also allows a quantitative scoring of the NPQ components. Fig. 2(e) visually demonstrates that the amplitude of the different NPQ components in the *F*_m_′ response increases as the corresponding data point moves away from the origin. For example, the *Pop‐qT* population extends along the q̃T axis, with points further from the origin showing a more significant increase in *F*_m_′ at light onset and relaxation to the basal level in the dark. Similarly, the *Pop‐qE* population exhibits a gradual increase in *F*_m_′ drop at light onset along the q̃E axis. We present the traces of the *Pop‐qI* and *Pop‐0* training populations for comparison and provide the evolution of the *F*_m_′ trace along the q̃I axis in Fig. S14. We also confirmed that the quantification of the NPQ components based on our machine learning framework matched that based on *ad hoc* metrics used on bulk populations (Allorent *et al*., 2013) (Fig. S15).

### Cell‐to‐cell variations in qE and qT

We used the 3D projection of traces to assess the cell‐to‐cell dispersion of *Pop‐qT* and *Pop‐qE* populations along the q̃T and q̃E axes, respectively. First, we conducted additional experiments to distinguish biological variations from experimental noise (fluorescence noise, instrumental noise, variations of incident light in the field of view, sample heterogeneity or imprecise dictionary reconstruction). While the training dataset consisted of synchronized nonmonoclonal populations, we performed new experiments with synchronized monoclonal populations of untreated *wt4a* ^*−*^ (*Pop‐qT*) and HL‐treated *stt7‐1* mutant (*Pop‐qE*) to minimize genetic diversity and the contribution of cell cycle to intercellular variations (see the Materials and Methods section). To assess noise level, we exploited our microscope setup, which allows us to apply the illumination protocol to the same cell maintained in the same position. We estimated that the distance in the 3D NPQ space between projections of the same cell under two consecutive repeats would provide a good estimate of the experimental noise, assuming the HL stress response remains consistent between the two repeats. Therefore, we compared the distribution of pairwise distance between two consecutive repeats on the same cell (*D*_*i*,*i*_) to that between all the other cells in the repeat (*D*_*i*,*j*_). If the distance distributions of *D*_*i*,*i*_ and *D*_*i*,*j*_ are similar, this suggests that experimental noise governs the scatter of the points cloud. Conversely, if the distribution of *D*_*i*,*j*_ exceeds that of *D*_*i*,*i*_, this would indicate that the scatter of the point clouds is mainly due to cell‐to‐cell biological variations.

First, we observed that the distributions of NPQ scores after two consecutive repeats were statistically similar (Fig. 3a,d), indicating that the average response to HL remained identical (see statistical approach in Fig. S16; Table S3). We then demonstrated that the distance distribution was twice as narrow for *D*_*i*,*i*_ as for *D*_*i*,*j*_ (Fig. 3b,e; a statistical test validated that the distribution variances are different, Table S3). Thus, we concluded that the scatter of the cloud points is mainly dominated by intercellular biological variations (see the Discussion section).

The distributions displayed in Fig. 3(a,d) indicate that the variation coefficients (standard deviation relative to the mean) are *c*. 40% and *c*. 20% for q̃E in *Pop‐qE* and q̃T in *Pop‐qT*, respectively. Interestingly, the variation coefficients are not statistically different from those of the nonmonoclonal synchronized algae for the reference dataset (Fig. 3c,f, Table S3), which supports that the distribution is not dominated by a genetic diversity. We hypothesized that heterogeneity in cell morphology or in the abiotic environment (e.g. variations in light intensity or in O_2_/CO_2_ availability associated with cell position in the sample) might contribute to the observed variability in photosynthesis. However, neither cell size (estimated from the projected cell area in the field of view) nor the cell's position within the field of view accounted for the variance (Fig. S17G–I).

### Projection of wild‐type traces in the 3D NPQ space

We then applied our approach to populations expressing both qE and qT by analyzing the projection of single‐cell *F*_m_′ traces from the HL‐treated WT *wt4a* ^*−*^ in 3D space. First, the monoclonal population *wt4a* ^*−*^ was HL‐treated for 4 h to induce the expression of qE, in addition to constitutively expressed qT. The average reconstruction error with the dictionary atoms was close to the selected threshold (2.5 × 10^*−*4^ for 77 cells), validating the hypothesis that the WT response can be accounted for by a combination of the training data. Then, we verified that experimental noise contributed minimally to the dispersion of the point cloud. Fig. 3(g,h) indicate that successive repeats of the HL protocol on the same cell showed similar mean scores for qE and qT. Moreover, the *D*_*i*,*i*_ distribution was narrower than *D*_*i*,*j*_ as previously observed (Fig. 3i), confirming once again that the dispersion is dominated by intercellular biological variations. The mean qE score was lower than that of the untreated *stt7‐1* population, consistent with the lower expression of qE in the WT compared to *stt7‐1* mutant as reported in the literature (Bonente *et al*., 2011; Bergner *et al*., 2015; Nawrocki *et al*., 2020). However, the population exhibited similar coefficients of variation of qE and qT to those observed in training populations expressing only one trait (43% for qE, 21% for qT in Fig. 3g,h). This significant level of heterogeneity in both NPQ components allowed us to exploit the natural intercellular variations to study the possible interaction between them.

We analyzed how intercellular variations evolve over the qE activation process by applying three sequential HL treatments of 80 min each, with measurements of *F*_m_′ traces after each step, leading to a total of four point clouds in the 3D space. The time evolution of the point clouds in the (q̃T, q̃E) plane is presented in Fig. 4(a). Fig. 4(b) demonstrates that our machine learning framework successively provides quantitative estimates of qE and qT from the complex *F*_m_′ traces (Fig. 1a), properly reconstructed by the dictionary method (Fig. S13). Fig. 4(c) shows the evolution of the average (±SD) of qE and qT scores, similar to what a bulk measurement would provide. The progressive increase of q̃E scores with HL exposure time (Fig. 4a,c) aligns with reported results showing that few hours of exposure to HL are needed to reach a steady qE level (Allorent *et al*., 2013). Surprisingly, we also observed a progressive decrease in q̃T scores (Fig. 4a,c). The alignment of linear (more correctly affine) fits in Fig. 4(a) demonstrated that qE and qT are strongly correlated, both at the centroids of the scatter plot and among individual cells treated with HL for 80, 160 or 240 min (Fig. 4a), confirmed by statistical tests (Table S3). This observation supports a potential synergistic action of qE and qT in response to HL (Roach & Na, 2017; Steen *et al*., 2022; Grossmann & Wollman, 2023). Although qI is not included in this plot, we verified that it does not affect the interplay between qE and qT. In fact, whether we examine the subpopulation of cells with the highest qI values or those with the lowest, the same correlation between qE and qT is observed (Fig. S18).

We performed the same experiments with another WT strain, *cc124* and found similar results at the population level (Fig. 4d,f). However, unlike *wt4a* ^***−***^, the correlation observed at the population level (point cloud centroids) is not reflected among individual cells of the same population (Fig. 4d). The *F*_m_′ traces displayed in the grid (Fig. 4e) were yet properly reconstructed by the dictionary method (Fig. S12).

## Discussion

### Comparison to established protocols and quantification of NPQ components

In this work, we used a combination of strains and treatments to generate elementary traces associated with each NPQ component. If the choice of the *stt7‐1* mutant to generate traces without qT was obvious, it was more delicate to find appropriate strains and conditions to eliminate qE. To create conditions without qE, we decided to exploit the property that LHCSR proteins are not expressed in a constitutive manner. This choice proved correct since the typical signatures of qE (rapid decrease and increase of *F*_m_′ at light onset and offset, respectively) were absent in the *Pop‐qI* and *Pop‐qT* populations, which were used to define the qI and qT axis respectively and were not expected to display qE (Fig. 1). Conversely, the qE signature was clearly observed in the *npq4* strain after HL treatment (Fig. S4), a characteristic previously associated with the presence of the second LHCSR protein, LHCSR1 (Peers *et al*., 2009; Girolomoni *et al*., 2019). The population *Pop‐0* is the weak point of our reference dataset, as it presents slight signatures of qE and qI. We made sure our protocol on the new instrument matched results from the literature (Fig. S15; reference Allorent *et al*., 2013).

Our framework requires an initial training phase with a representative population for each phenotype. This limits its application to species for which the necessary resources and expertise are available to build such an informed training dataset. In fact, we experienced this drawback when including the contribution of qI in our analysis, for which the underlying biological processes are not as well controlled and informed as for qE and qT. We see however in (Ramakers *et al*., 2025) that an alternative unsupervised approach can have difficulties to extract generic signatures across wild types and mutants.

One may methodologically question our strong working hypothesis which implies that for every *F*_m_′ trace, the kinetics of each NPQ contribution (e.g. qE) will be reflected somewhere in the collection of single‐cell *F*_m_′ traces contained in the population representing this NPQ contribution (e.g. *Pop‐qE* for qE) in the training dataset. Here, the intermediate reconstruction step with Dictionary Learning assesses the quality of the reconstruction of the *F*_m_′ traces by an average mean‐squared error test and allows to accept or reject the hypothesis. Failure to reconstruct the traces within the training error margin either invalidates this assumption, or indicates an additional NPQ component not covered by the training dataset. We showed that the *F*_m_′ trace of strains exhibiting several NPQ components is effectively captured by a framework built exclusively on populations expressing a single NPQ component (Fig. S13). As shown in Fig. S13, we have confirmed that this hypothesis is applicable to a wide range of strains that were not included in the training dataset and with different genetic background.

We confirmed that the NPQ scores generated by our machine learning framework were consistent with the *ad hoc* measurements commonly used in the literature (Figs 2, S15), demonstrating the ability of our agnostic method to relate to hand‐crafted metrics, offering potential beyond our case study.

### Quality check of the cell‐to‐cell heterogeneity

We were interested in studying the cell‐to‐cell heterogeneity of responses to light‐stress. We validated our analysis framework to ensure low biases and noise in single‐cell responses, preserving our interpretation of intercellular variability or trait correlations through key quality checks that we believe should be reproduced in future works inspired by our approach.

- *Validation of the instrument*. We validated our new instrument and protocols by comparing the results obtained with a classical instrument (Fig. 1d,e). We confirmed the absence of bias related to light heterogeneity or the position of the cell in the field of view (Fig. S17).
- *Assessment of the noise impact*. We conducted two successive experiments with each cell assumed to be in an identical state, analyzed the cell‐to‐itself and cell‐to‐population response variances to evaluate the experimental noise (Fig. 3b,e,i). Achieving identical behavior across all cells in repeated experiments is an unattainable goal, so the noise levels are likely overestimated, which reinforces our conclusions.
- *Reduction of identified sources of biological variation*. Given the reported causal relationships between cell size and photosynthetic activity (Malerba *et al*., 2018), we confirmed that the NPQ score was not influenced by cell size (Fig. S17D–F). Another potential source of variability is related to the cell cycle (Cross & Umen, 2015). To minimize this source of variability, we used cultures of cells synchronized on a 12 h : 12 h, light : dark cycle (see the Materials and Methods section; Fig. S7) and performed experiments at the same time of day (*c*. 2 h after the beginning of the light phase). Monoclonal cultures were used when needed to minimize the risk of genetic heterogeneity.

Although we cannot fully exclude other sources of heterogeneity introduced during the cell preparation before measurements, we conclude that the observed variability in qE (coefficient of variation *c*. 0.4) and qT (coefficient of variation *c*. 0.2) likely reflects stochastic gene expression (McAdams & Arkin, 1997; Elowitz *et al*., 2002).

### Molecular origins of the strong correlation between qE and qT

The substantial intercellular variability of qE and qT allowed to identify a strong negative correlation between qE and qT in synchronized monoclonal cultures of *wt4a* ^***−***^ (Fig. 4a). This result is particularly significant because it emerges within the same genotype and cellular context, among cells with common histories. The correlation can be summarized as follows: the higher the qE under HL, the less pronounced the transition from state II to state I.

**Fig. 5:**
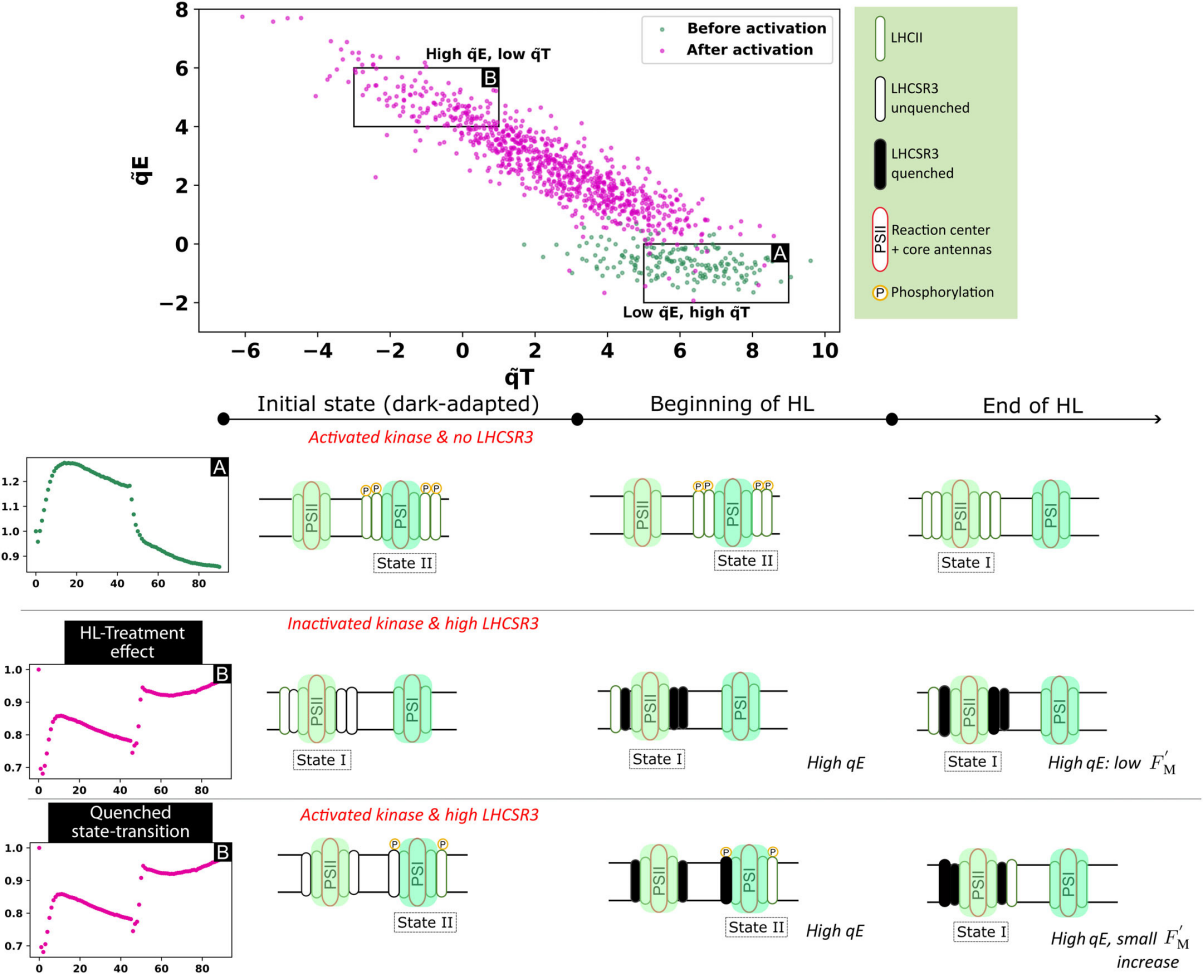
Two tentative hypotheses accounting for the observed correlation between qE and qT: high‐light treatment (HL treatment) effect and quenched state transition. The graph corresponds to Fig. 4(a), showing the evolution of qE and qT before (green) and after HL treatment of 80, 160 and 240 min combined (red) of the strain *wt4a* ^*−*^. The ‘initial state (dark‐adapted)’ column represents the initial state of the dark‐adapted sample (first *F*_m_′ at the beginning of the light protocol). The ‘beginning of HL’ column represents the situation after less than 1 min of HL (first *F*_m_′ after the onset of HL). The ‘End of HL’ column represents the situation at the end of the HL period (last *F*_m_′ before offset of light). Case A represents *F*_m_′ traces before activation (low qE and high qT) while case B shows *F*_m_′ of activated cells representative of high qE and low qT. See text for a description of the hypotheses.

There are several potential links between qE and qT, already discussed previously (Allorent *et al*., 2013), which allow to identify tentative hypotheses that are not mutually exclusive and can together explain the strong correlation between qE and qT in the HL‐treated *wt4a* ^***−***^ (Fig. 5). First of all, it has long been established that exposure to HL triggers both the induction of the LHCSR3 genes, which drive qE (Peers *et al*., 2009; Allorent *et al*., 2013), and the inactivation of STT7 involved in qT (Rintamäki *et al*., 2000; Vink *et al*., 2004). The progressive decline in qT scores over time under HL exposure – attributed to an increasing proportion of cells locked in state I – and the concomitant increase in qE are therefore unsurprising. However, this population‐level response does not explain the alignment of qE/qT correlations in the *wt4a* ^*−*^ *WT* when comparing the centroids of the scatter plots at different times of HL exposure or all individual cells within a given time point (Fig. 4a). These findings point to a more intricate interaction between qE and qT than cannot be inferred from bulk measurements, which obscure such details through population‐level averaging.

Although exploring the molecular basis of this correlation is outside the scope of this study, we would like to propose a few tentative hypotheses. One possibility is that common HL signaling pathways – such as photosyntheticretrograde signals including the redox state of the plastoquinol/plastoquinone pool or reactive oxygen species – are coupled, leading simultaneously to reduced STT7 activity and to increased LHCSR3 amounts. In this scenario (Fig. 5; ‘HL treatment effect’), cells with higher LHCSR3 would also exhibit a more pronounced reduction in the accumulation or activity of STT7, irrespective of HL exposure time.

Alternatively, a mechanistic coupling between qE and qT could exist, where, for the same amounts of LHCSR3 or STT7, the extent of state transitions influences heat dissipation in PSII, or vice versa. An obvious mechanistic coupling between the two processes arises from the fact that, for a given amount of LHCII transferred from PSI to PSII, the resulting fluorescence increase will be smaller (lower qT score) if PSII fluorescence is strongly quenched (higher qE score). In this ‘quenched state‐transitions’ hypothesis (Fig. 5), an initial cell‐to‐cell heterogeneity in the amount of LHCSR3 bound to PSII (reflecting variability in qE capacity) leads to a corresponding variation in fluorescence increase for each LHCII complex moving from PSI to PSII. The reported phosphorylation of LHCSR proteins by STT7 could also participate in this coupling (Bonente *et al*., 2011; Allorent *et al*., 2013; Bergner *et al*., 2015). Other potential mechanistic links may involve cyclic electron flow (CEF) around PSI or the activation state of the CBB cycle.

Regardless of the molecular causes for the different behaviors of the *wt4a* ^*−*^ and *cc124* WTs, their comparison provides two insights. First, it highlights the value of single‐cell studies, as two populations may orchestrate their responses to HL differently, while displaying similar behavior at the macro (population) level. Second, it highlights the potential influence of the Yule–Simpson paradox in photosynthesis research, where a correlation observed at the population level may not reflect individual causal relationship (Simpson, 1951).

### Perspectives for single‐cell photosynthesis measurements

We demonstrated that our machine learning framework is able to predict stress‐response levels without *a priori* knowledge of the temporal phases, but taking into account only the dynamics of the signal. As the framework is agnostic to the biological question, it can be readily extended to other single‐cell phenotyping applications involving kinetic responses, across different species and stress types. For instance, it could be adapted to study responses to nutrient deficiencies or extreme temperatures, provided that these stresses trigger detectable changes in fluorescence yields.

Importantly, this single‐cell machine learning approach allows us to derive information on stress response heterogeneity within populations with a small number of experiments. It also allows for the identification of statistically significant correlations between different stress responses, highlighting an underlying biological interplay. One promising application is the study of the interplay between PsbS‐ and LHCSR3‐dependent NPQ components in mosses. Indeed, because mosses offer powerful genetic tools and occupy a key evolutionary position, they are ideal model organisms for studying the evolution of plant photoprotection, especially the distinct and overlapping roles of PsbS and LHCSR3, which they uniquely conserve (Gerotto *et al*., 2011; Gao *et al*., 2022). Other applications could explore interactions between photosynthetic traits related to photochemical quenching rather than those related to NPQ components, and from there, study the interconnected network of alternative electron flows operating in parallel with linear electron transport. The robustness of photosynthesis relies on this network and a particularly elegant example of reciprocal compensation is that between the CEF and the flavodiiron pathway in *C. reinhardtii* (Burlacot *et al*., 2022). Provided the method is sensitive enough under lower light intensities than those typically used to probe NPQ, our approach could, for example, be applied to facilitate an in‐depth investigation of their interaction.

Future work could integrate ChlF measurements with flow cytometry/microfluidics and/or omics methods to correlate cell morphology and photosynthetic traits, facilitating applications such as varietal selection, directed evolution and single‐cell screening.

## Competing interests

None declared.

## Author contributions

AL, DC, LJ, TLS and BB were involved in conceptualization. AL, MO, SB, PH, DC, LJ, TLS and BB were involved in methodology. AL, WG, PH and DC were involved in software. AL, MO, SB, DC and BB were involved in validation and formal analysis. AL, MO, SB, EI, PH, DC, LJ, TLS and BB were involved in resources and investigation. AL, MO, WG and DC were involved in data curation. AL, LJ, DC and BB were involved in writing – original draft. AL, MO, SB, WG, EI, PH, DC, LJ, TLS and BB were involved in writing – review and editing. AL, MO, SB, WG, DC and BB were involved in visualization. AL, PH, DC, LJ, TLS and BB were involved in supervision. LJ, TLS, PH, DC and BB were involved in project administration and funding acquisition.

## Disclaimer

The New Phytologist Foundation remains neutral with regard to jurisdictional claims in maps and in any institutional affiliations.

## Supporting information


**Fig. S1** Description of the optical setup.
**Fig. S2** Light source spectra.
**Fig. S3** Calibration of the light sources.
**Fig. S4** Investigation of HL‐treatment in *npq4* strain.
**Fig. S5** Effect of lincomycin treatment on the slow nonphotochemical quenching component.
**Fig. S6** Image of the agarose pad used for single‐cell measurements.
**Fig. S7** Evaluation of the synchronicity within the Chlamydomonas population.
**Fig. S8** Watershed segmentation of the fluorescence movies.
**Fig. S9** Description of the reference protocol illumination.
**Fig. S10** Evaluation of the quenching effect of the saturating pulse.
**Fig. S11** Full excitation sequence allowing to probe qE, qI, qT, activate qE and achieve a negligible contribution of qI.
**Fig. S12** Exploration of the role of the hyperparameters of the dictionary learning on the reconstruction error.
**Fig. S13** Dictionary learning reconstruction for phenotypes absent from the training set.
**Fig. S14** Qualitative analysis of the evolution of the fluorescence traces along the q̃I and q̃T axis.
**Fig. S15** Comparison of the nonphotochemical quenching features identified in the literature with the metrics extracted with the machine learning pipeline.
**Fig. S16** Distribution of the nonphotochemical quenching scores for the two consecutive repeats (third and fourth) of the experiments on monoclonal populations.
**Fig. S17** Investigation of the correlation of the nonphotochemical quenching score with various parameters.
**Fig. S18** Correlation between q̃E and q̃T in subpopulations of wild‐type *wt4a* ^−^ with low and high q̃I.
**Table S1** Illumination equipment.
**Table S2** Summary of the light protocols.
**Table S3** Statistical tests performed on the elements presented in the main text.
Please note: Wiley is not responsible for the content or functionality of any Supporting Information supplied by the authors. Any queries (other than missing material) should be directed to the *New Phytologist* Central Office.

## Data Availability

The data that support the findings of this study are openly available in NPQScore‐data folder dataset.zip at doi: 10.5281/zenodo.14606011. The repository is also hosted on Github (https://github.com/DreamRepo/NPQScore‐data) and sample codes to access the data and reproduce the results are provided.
