# Supplementary material for: Interplay between high‐energy quenching and state transitions in Chlamydomonas reinhardtii: a single‐cell approach
Source: New Phytol. 2026 Mar 22;250(4):2689–705. doi: 10.1111/nph.71001 (PMC13103431; doi:10.1111/nph.71001)
Supplement: Supplementary file 1 — Fig. S1 Description of the optical setup. Fig. S2 Light source spectra. Fig. S3 Calibration of the light sources. Fig. S4 Investigation of HL‐treatment in npq4 strain. Fig. S5 Effect of lincomycin treatment on the slow nonphotochemical quenching component. Fig. S6 Image of the agarose pad used for single‐cell measurements. Fig. S7 Evaluation of the synchronicity within the Chlamydomonas population. Fig. S8 Watershed segmentation of the fluorescence movies. Fig. S9 Description of the reference protocol illumination. Fig. S10 Evaluation of the quenching effect of the saturating pulse. Fig. S11 Full excitation sequence allowing to probe qE, qI, qT, activate qE and achieve a negligible contribution of qI. Fig. S12 Exploration of the role of the hyperparameters of the dictionary learning on the reconstruction error. Fig. S13 Dictionary learning reconstruction for phenotypes absent from the training set. Fig. S14 Qualitative analysis of the evolution of the fluorescence traces along the q̃I and q̃T axis. Fig. S15 Comparison of the nonphotochemical quenching features identified in the literature with the metrics extracted with the machine learning pipeline. Fig. S16 Distribution of the nonphotochemical quenching scores for the two consecutive repeats (third and fourth) of the experiments on monoclonal populations. Fig. S17 Investigation of the correlation of the nonphotochemical quenching score with various parameters. Fig. S18 Correlation between q̃E and q̃T in subpopulations of wild‐type wt4a − with low and high q̃I. Table S1 Illumination equipment. Table S2 Summary of the light protocols. Table S3 Statistical tests performed on the elements presented in the main text. Please note: Wiley is not responsible for the content or functionality of any Supporting Information supplied by the authors. Any queries (other than missing material) should be directed to the New Phytologist Central Office. [file NPH-250-2689-s001.pdf]

New Phytologist Supporting Information

Title: Interplay between high-energy quenching and state transitions in *Chlamydomonas reinhardtii*: a single-cell approach.

Authors: Aliénor Lahlou[1,2*], Marcelo Orlando[3], Sandrine Bujaldon[3], William Gaultier[2], Eliora Israelievitch[3], Peter Hanappe[2], Thomas Le Saux[1], Ludovic Jullien[1], David Colliaux[2*], Benjamin Bailleul[3,4*]

[1]CPCV, Department of Chemistry, École Normale Supérieure, PSL University, Sorbonne University, CNRS, Paris, France
[2]Paris Research, Sony Computer Science Laboratories, Paris, France
[3]UMR7141: Photobiology and Physiology of Plastids and Microalgae, Institut de Biologie Physico-Chimique, Sorbonne University, CNRS, Paris, France
[4]4. UMR7144 : Adaptation and Diversity in the Marine Environment (ECOMAP), Station Biologique de Roscoff, Sorbonne University, CNRS, Roscoff, France

Article acceptance date: 8 January 2026

Contents

1 Description of the fluorescence microscope **3**
1.1 Light part . . . 3
1.2 Imaging part . . . 4
1.3 Heterogeneity of illumination . . . 5
1.4 Instrument control . . . 5

2 Biological samples **5**
2.1 Investigation of the strain *npq*4 . . . 5
2.2 Evidence of a slow relaxing NPQ component . . . 7
2.3 Growth of algae on the pad . . . 7
2.4 Algae synchronization . . . 7
2.5 Image analysis of the single-cell Chlorophyll fluorescence movies . . . 8
2.6 Analysis of polypeptides . . . 10

3 Data collection **11**
3.1 Illumination protocols . . . 11
3.1.1 Protocol inducing high-light stress and used to collect $F_m^{'}$ (reference protocol) . . . 11
3.1.2 Summary of the light protocols . . . 11
3.2 Evaluation of the quenching effect of the saturating pulses . . . 12
3.3 Generation of the F_m' traces . . . 12

4 Dictionary learning to represent the F_m' traces **15**
4.1 Investigation of the hyperparameter space . . . 15
4.2 Validation of the reconstruction on F_m' traces . . . 16

5 Linear Discriminant Analysis (LDA) to project the dataset onto the NPQ space **16**

6 Comparison with *ad hoc* metrics **19**

7 Statistical tests **20**
7.1 Monoclonal experiments 20
7.2 Main text figures 20

8 Sources of heterogeneity **22**

9 Evaluation of the relevance of the axis $\tilde{q}$I **23**

1 Description of the fluorescence microscope

The home-made fluorescence microscope used for the experiments is pictured in Figure S1. In the following we present the technical choices for the illumination and the imaging parts, and the instrument controls. We built the microscope with Thorlabs parts, in three stages combined with thick Thorlabs posts to maximize the stability of the setup.

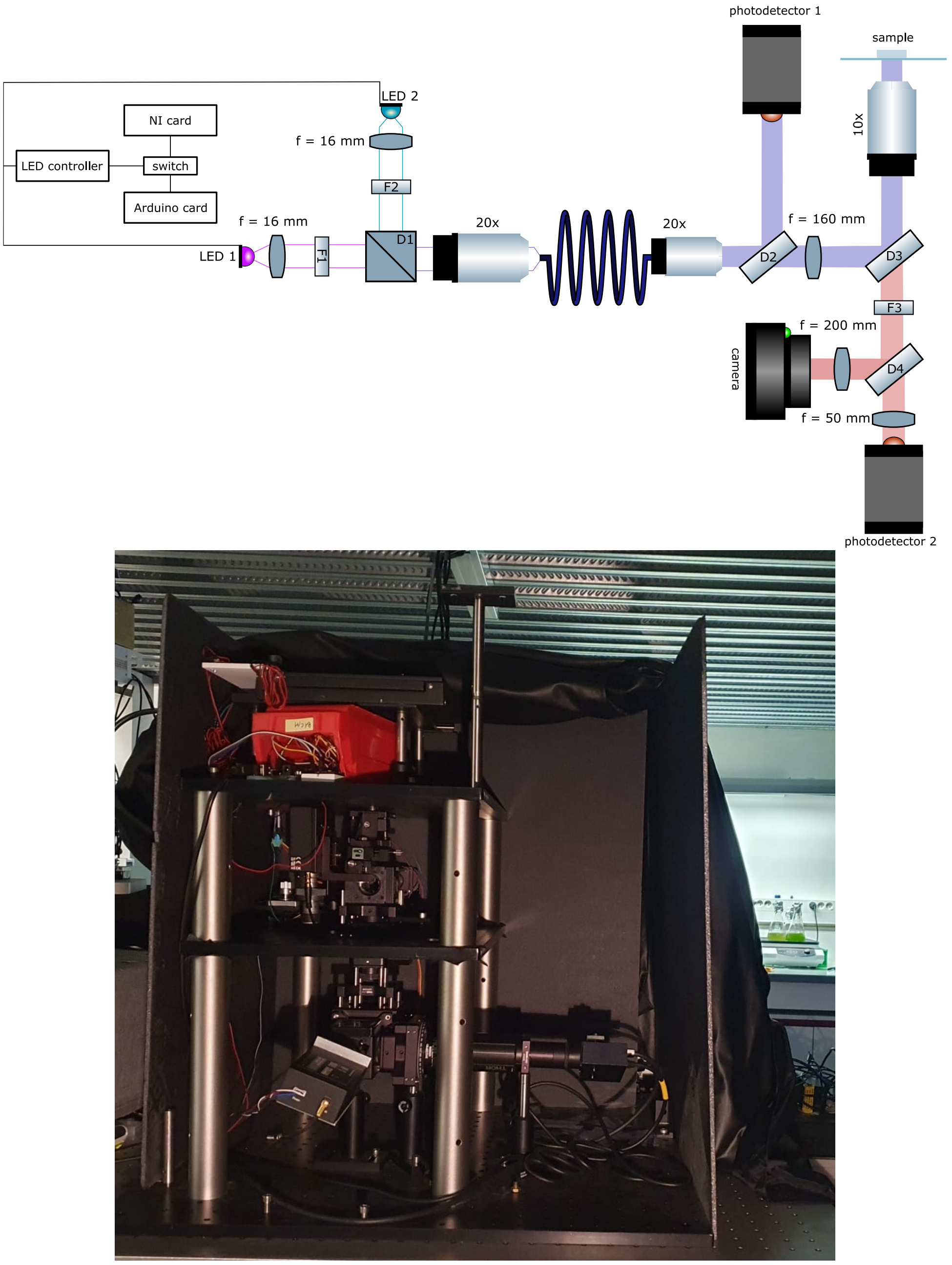

Figure S1: Description of the optical setup. Top: Scheme of the optical setup. LEDs and band filters: 1: 405 nm, 2: 470 nm; Dichroic high-pass filters: D1: 425 nm (to combine 405 nm and 470 nm), D2: glass plate to reflect incident light on detector, D3: 506 nm (to send light excitation towards the sample and collect the fluorescence), D4: 699 nm (cuts-off in the middle of the ChlF spectrum to split the signal between the photodetector and the camera - can be removed or replaced by a mirror); Bottom: Picture of the setup.

1.1 Light part

The five light sources (Table S1) are high-power LEDs mounted on cages (*CXY1 - Thorlabs*). They are collimated by lenses with a focal distance of 16 mm (*ACL25416U - Thorlabs*) then filtered by band-pass filters (Table S1). The lights are combined by dichroic filters (Table S1) to be merged and injected into an optical fiber ($\emptyset = 400\ \mu m, NA = 0.5$ - *M45L02 Thorlabs*) through a microscope objective (*Plan APO 20X/0.75 DIC N2 - Nikon*). A filter wheel (*FW102C - Thorlabs*) is set in front of the blue LED allowing to explore a range of intensity

from 1 μmol(photons) $\cdot$ m^{-2} $\cdot$ s^{-1}to 10000 μmol(photons) $\cdot$ m^{-2} $\cdot$ s^{-1}. By "intensity" we refer to "photon irradiance", corresponding to the number of photons crossing a unit surface area per unit time [1]. The LEDs are controlled by a voltage to intensity converter (*DC4100 - Thorlabs*). It can power four LEDs and is controlled by four voltage inputs. Depending on the type of signal expected, a different controller is used to send the voltage inputs, and a switch allows to keep the signals into two separate circuits. A National Instruments Data Acquisition Card *SCB68A* with *PCI 6374* generates time signals written as Python arrays, or an *Arduino Uno* controller generates precisely-timed pulses of light. The spectra of the blue and violet filtered LEDs used in this work have been acquired on a LPS 220 spectrofluorometer (PTI, Monmouth Junction, NJ) and correspond to 470±10 nm and 405±7 nm(Figure S2).

name	quality	wavelength	reference	supplier
L1	high power LED	violet	LHUV0-405-A065	Lumileds
L2	high power LED	blue	LXZ1-PB01	Lumileds
F1	band-pass filter	405/20	ZET405/20x	Chroma
F2	band-pass filter	479/40	FF01-479/40-25	Semrock
F3	band-pass filter	775/140	FF01 775/140	AHF
F3bis	band-pass filter	675/90	675/90 ET	AHF
D1	long-pass dichroic filter	425	t425lpxr	Chroma
D2	glass microscope plate	NA		
D3	long-pass dichroic filter	506	FF506-Di03	Semrock
D4	long-pass dichroic filter	699	FF699-FDi01	Semrock

Table S1: Illumination equipment.

1.2 Imaging part

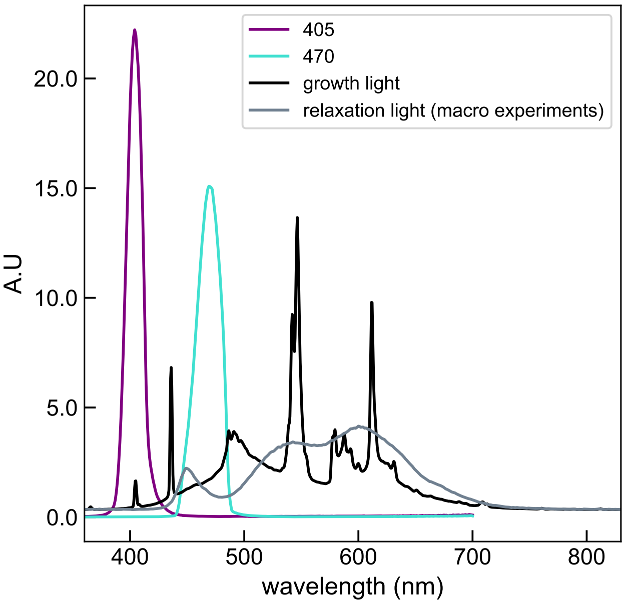

Figure S2: Light source spectra. the spectra of the blue and violet light sources were measured with a fluorometer and normalized by their average. We also provide the spectra of the growth light, as well as relaxation light used for macro experiments. They were measured with a BLUE-Wave miniature spectrometer (*StellarNet, US*).

The optical fiber output is connected to a microscope objective (*Plan 10x/.25 - Olympus*). A fraction of the illumination flux is reflected towards a photon counter by a microscope glass plate D2 installed on the light path. It allows to monitor the intensity of the LEDs for a given voltage input with Photodetector 1 (*Multi-Pixel Photon Counter C13366 - Hamamatsu*). The light path crosses a tunable diaphragm and a lens (*focal length 150 mm, AC254-150-A-ML - Thorlabs*). A dichroic filter D3 (Table S1) reflects the excitation light towards a microscope objective (*Objective Fluar 10x-0.50-M27 - Zeiss*). The objective can easily be changed for another magnification, with a flexibility in the working distance in the range of several centimeters, thanks to Thorlab rings stacking. The objective is mounted on a plate

that moves in the z-direction to control the focus (*NFL5DP20/M - Thorlabs*). The plate can also be controlled by a piezoelectric (*KPZ101 - Thorlabs*, and we added the possibility to use a motor controlled by an Arduino to move the stage with a coarser precision[1]). The diaphragm is conjugated with the microscope plane and allows to reduce the diameter of the excitation light, and excite a limited area in the sample. The fluorescence is collected by the same microscope objective and is transmitted by the dichroic filter D4 (Table S1) and a fluorescence filter F3 (Table S1) towards the measuring devices. The fluorescence light can be either collected on a camera (*Ueye 3060CT-M - IDS*) conjugated with a tube lens (2 inches, focal length 200 mm, to maximally collect the photons), or a Photodetector 2 (*Multi-Pixel Photon Counter C13366 - Hamamatsu*) conjugated with a lens of focal length 50 mm. We can also collect the signal on both, depending on the filter used: mirror, dichroic filter D8 whose cut-off is in the middle of the ChlF spectrum (Table S1), or no filter. The camera has adaptable gain and exposure time and is triggered internally or externally depending on the experiment. Unless specified, the camera was neither binned nor sub-sampled and the image size is 1936×1216 pixels[2].

1.3 Heterogeneity of illumination

To study the homogeneity of the field of illumination, we performed the calibration process using Dronpa-2 solution as described in [2]. We analyzed the temporal response of the fluorescence of **Dronpa-2** extracted from the movie. Figure S3a shows a frame of the movie where the blue and violet LEDs are ON, while Figure S3b shows a slide where only the blue LED is on, evidencing the decay of fluorescence. The video (608×968 pixel^2) was downsized by a 3×3 averaging kernel to improve signal-to-noise ratio. The 470 LED was kept constant and the 405 one was switched ON and OFF four times for 10 s every 24 s. The first response was discarded due to the difference of response of the dark state of Dronpa-2.[3] The mask of the illuminated area was evaluated with a threshold on the intensity image and the following operations were performed only on the unmasked pixels. Monoexponential fits were performed on each ON-OFF and OFF-ON phase and the retrieved characteristic times were averaged. From the extracted characteristic times, the light intensity in μmol(photons) $\cdot$ m^{-2} $\cdot$ s^{-1}was computed for each pixel using the molecular action cross-sections for photoisomerisation of Dronpa-2 [2]. The mean value of light intensity in the field in the given conditions is 8.0 and 1.4 μmol(photons) $\cdot$ m^{-2} $\cdot$ s^{-1}for the 470 and 405 LEDs respectively. The conditions chosen for these experiments are optimal for computing the distribution map with high resolution. In the experiments with algae, an optical density filter is placed in front of the 470 LED, before the injection in the optical fiber. The map of intensities derived from the relaxation times of the fluorescence evolution for the blue LED and the violet LED are shown in Figure S3c,d. The distribution of intensities presents a narrow distribution (Figures S3e,f). There is a 16% variation of light intensity in the field of view for the blue actinic LED and 30% variation for the violet SP LED.

1.4 Instrument control

The automated instruments (filter wheel, $x - z$ displacements, LED controller, LED switch) are controlled by serial communication with Python codes. The excitation signals are sent either through an Arduino Uno microcontroller or a DAQ National Instruments card (*SCB68A* with *PCI 6374*). The fluorescence signals are acquired through a camera (*UEye 3060CT-M - IDS*) controlled with Python or through the MPPC signals collected with the DAQ card. An autofocus routine allows to adjust automatically the Z position to maximize the Laplacian variance of the image before each experiment, as described in [4].

For each experiment, the codes and the results are saved in a database MongoDB with the Python library Sacred.[2].

2 Biological samples

2.1 Investigation of the strain *npq*4

The mutant *npq*4 lacks the LHCSR3 protein and it is commonly used in the literature as a negative control for qE. We noticed that the mutant *npq*4 exhibited qE after 4 h of HL-treatment under the microscope on the agarose pad. As shown in Figure S4b the fluorescence

[1] https://github.com/SonyCSLParis/ControlMotors
[2] https://github.com/IDSIA/sacred

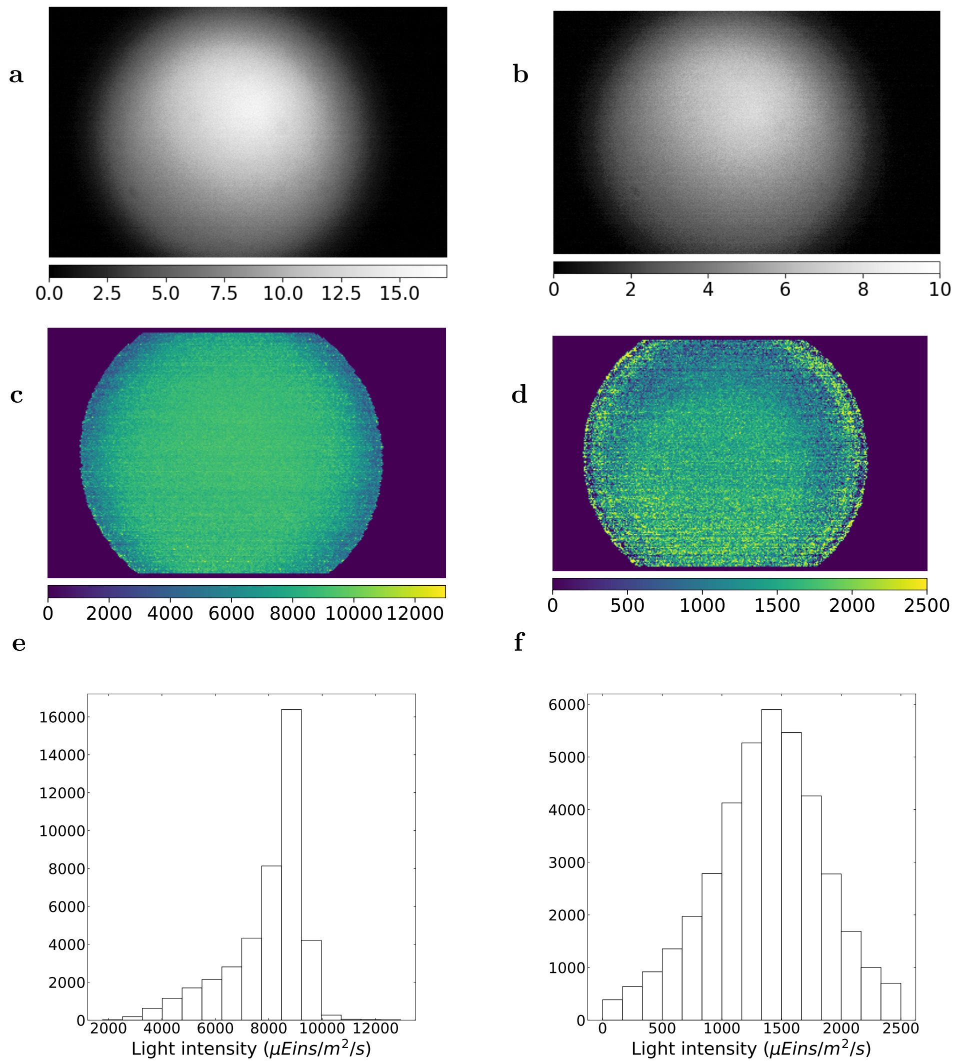

Figure S3: Calibration of the light sources. **a,b**: Intensity map of the 470 (resp. 405) LED in the focal plane calibrated with **Dronpa-2** for voltage inputs of the LED controller 500 mV (resp. 250 mV); **c,d**: Histogram of the intensity values, $(\mu, \sigma) = (8.0,1.3)$ (resp. (1.4,0.4) $mmol(photons).m^{-2}.s^{-1}$).

drops sharply at the light onset, characteristic of qE. The amplitude of the drop lies between the characteristic drops found in ***Pop__0*** (Figure S4a) and ***Pop__qE*** (Figure S4c). This behavior has previously been identified in the literature and attributed to an over-expression of LHCSR1 to compensate for the absence of LHCSR3[5, 6]. See however [7] for a different view on the function of LHCSR1.

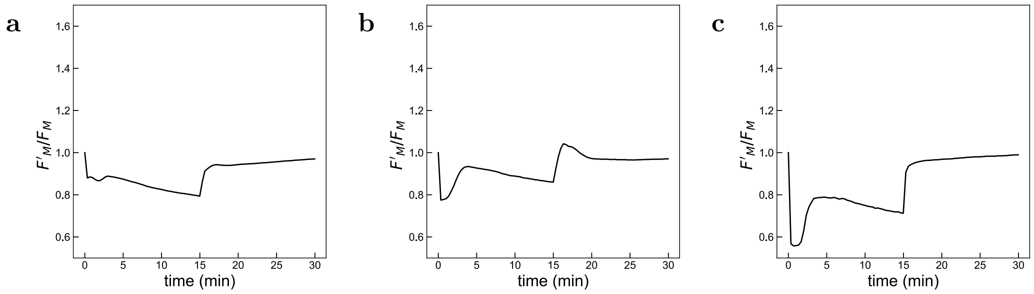

Figure S4: Investigation of High-Light-treatment in *npq4* strain. Maximal fluorescence yield F'_m normalized to the first point F_m averaged across the population of algae in an agarose pad observed under the fluorescence microscope (normalized to F_m). **a**: ***Pop__0*** (*stt7-1* harvested from growth light); **b**: *npq*4 after HL-treatment, fourth repeat of the reference protocol. **c**: ***Pop__qE*** (*stt7-1* after HL-treatement).

Rather than using a double-mutant of LHCSR1 and LHCSR3, which exhibits compensatory physiological reorganization [8], we chose to grow cells in low light in the presence of

acetate (see Methods). The CO_2 produced by the metabolism of acteate represses LHCSR3 which, combined with non-detectable levels of LHCSR1 in low light conditions, creates qE-free conditions [9].

2.2 Evidence of a slow relaxing NPQ component

We formulated the hypothesis that the slow NPQ component corresponds to photoinhibition. To test this, we used 1 mM of lincomycin, an inhibitor of D1 protein repair. Indeed, PSII photodamage and qI occur when excessive light disrupts the balance of the continuous degradation and repair process of reaction centers involving the D1 protein, encoded in the chloroplast genome [10, 11]. Experiments were conducted using the SpeedZen set-up, as described in the Methods, to evaluate population's response to high-light illumination and its relaxation in the dark. We applied the basic excitation pattern on the SpeedZen, exposing a culture of stt7-1 to high-light (740 μmol(photons) $\cdot$ m^{-2} $\cdot$ s^{-1}, 620 nm) for 15 min, followed by 15 min of dark relaxation, while measuring the evolution of F'_m with saturating pulses (SP).

In our approach, we suggest that after two consecutive illuminations with the reference protocol, the D1 protein repair mechanism is active and the slow NPQ mechanism reaches steady-state. We tested this hypothesis by adding lincomycin in the dark adapted population just before the first illumination, and also after applying the light protocol two times in a row, and comparing the F'_m traces to the control population without lincomycin.

In the first illumination (Figure S5a) we see no significant difference (P-value = 0.36) between the lincomycin-treated population and the control populations after 15 min of high light. However, at the end of the 15-minute dark relaxation we observe a significant (P-value = 0.001) but very small difference in the recovery. Suggesting that even without lincomycin, the D1 protein repair mechanism is only minimally active in the first illumination. After two consecutive illuminations (Figure S5b) we start noticing the presence of the fast NPQ component qE that is induced by the consecutive illuminations. But more importantly we see an increase in the slope of the slow NPQ component with the addition of lincomycin. Indeed, after 15 min of high light, F'_m/F_m is significantly higher (P-value = 10-7) for the control without lincomycin (0.726 $\pm$ 0.003) compared to the lincomycin-treated population (0.68 $\pm$ 0.01). As expected, we notice a greater effect after 15 min of relaxation in darkness, with F'_m/F_m values of 0.86 $\pm$ 0.01 for the control condition and 0.75 $\pm$ 0.01 for the lincomycin-treated sample (P-value = 10-8). These results support our hypothesis that by the third illumination, the D1 repair mechanism is fully active and compensates the high-light-induced PSII damage, leading to a reduced contribution of qI in the F'_m traces.

To test whether the small effect of lincomycin in NPQ recovery after the first illumination protocol is indeed related to photoinhibition, we repeated the experiment with 40 min of relaxation in darkness instead of 15 min. Expecting this extended period would allow sufficient time for the D1 protein repair mechanism to activate and for the slow NPQ component to relax in the control sample. We also spaced each SP in darkness by 1 minute, rather than 20 s, to minimize any HL stress associated to SP. As can be seen in Figure S5c, after 40 min of dark relaxation we measured a significant difference (P-value = 10-7) in recovery between the lincomycin-treated population (F'_m/F_m = 0.690 $\pm$ 0.004) and the control population without lincomycin (F'_m/F_m = 0.756 $\pm$ 0.004). Therefore, we consider that the slowly reversible quenching in our dataset is dominated by qI, although a contribution from qZ cannot be strictly excluded [12].

2.3 Growth of algae on the pad

We left the algae to grow with intermittent 15 min HL/15 min dark (HL: 400 μmol(photons) $\cdot$ m^{-2} $\cdot$ s^{-1}, 470$\pm$10 nm) for several days and showed the growth was not limited by the sample preparation (Figure S6a). In Figure S6b we show the algae after a first division. Figure S6c shows the same sample after four and a half day. The surface occupied by algae was multiplied by 14 between the two images.

2.4 Algae synchronization

To assess the quality of the synchronization described in Methods, we plotted the number of algae segmented in the field of view over the experiment time (segmentation method: Figure S8). Over a 20 h long experiment, we observed an initial plateau, a sharp increase in the cell

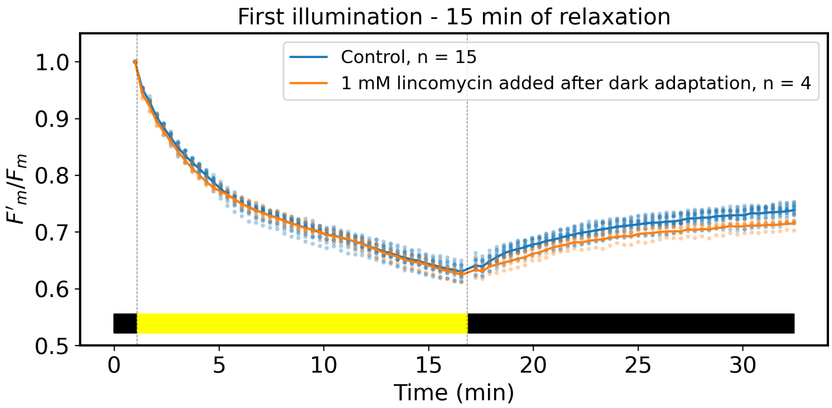

a

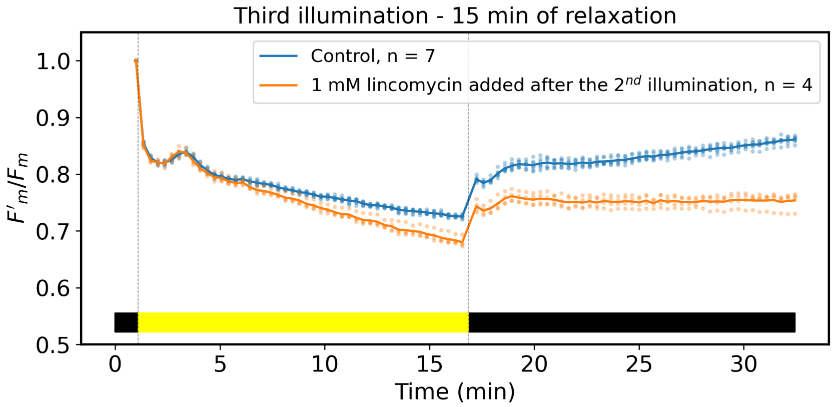

b

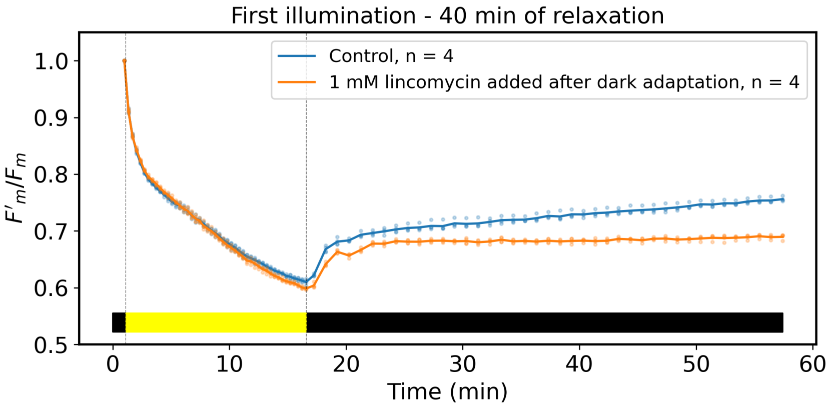

c

Figure S5: Effect of lincomycin treatment on the slow non-photochemical quenching (NPQ) component. Maximal fluorescence yield F'_m normalized to the first point F_m, averaged on a population of stt7-1 on the SpeedZen exposed to lincomycin either after dark adaptation (a) and (c) or after the second illumination of the protocol (b). The dots represent experimental data from different technical replicates, while the continuous lines connect the averages across the replicates for each time point. High-light: 740 μmol(photons) $\cdot$ m^{-2} $\cdot$ s^{-1}, 620 nm. Number of repeats: n (see legend).

number and another potential plateau (see Figure S7). When fitting with a sigmoidal curve the evolution of the number of algae, we found a characteristic time below 3h, demonstrating that a synchronous rhythm is imprinted in the algae division.

2.5 Image analysis of the single-cell Chlorophyll fluorescence movies

The algae in the video were segmented using local mathematical morphology operations described in *Chouket et al* [13] with the codes given in the corresponding Supplementary Materials [14] developed to segment fluorescent bacteria. As opposed to bacteria, algae are round and do not display a local maxima of fluorescence in the cell center. Instead, they show dark regions in the middle, corresponding to the part unoccupied by the fluorescent chloroplast. As a result, we changed the watershed source identification method: instead of locating local maxima of fluorescence, we locate the center of the algae. We begin by using a fill-holes operation to obtain only round and filled objects, which is not initially the case

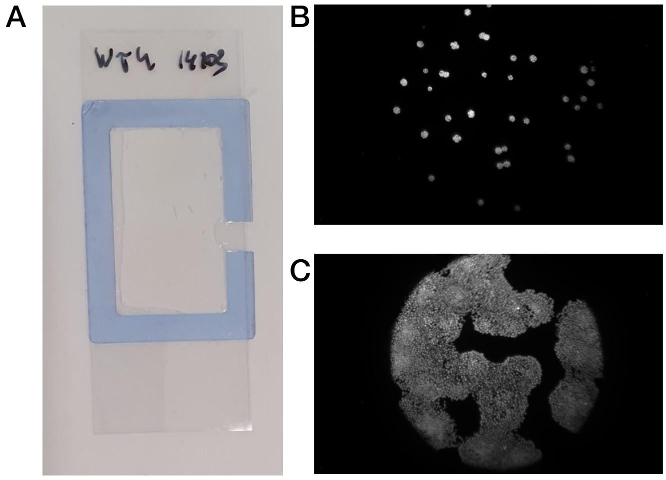

Figure S6: Image of the agarose pad used for single-cell measurements. **a**: Sample preparation: the medium mixed with agarose is spread on a pad and solidified. A drop of algae is deposited on the agar mix and covered by a microscope slide. **b,c**: Demonstration of the viability of the cells on the agarose pad. Fluorescence images of wild-type strain taken four and a half days apart. The surface occupied by the algae is multiplied by 14. Strain: *wt4a*$^-$.

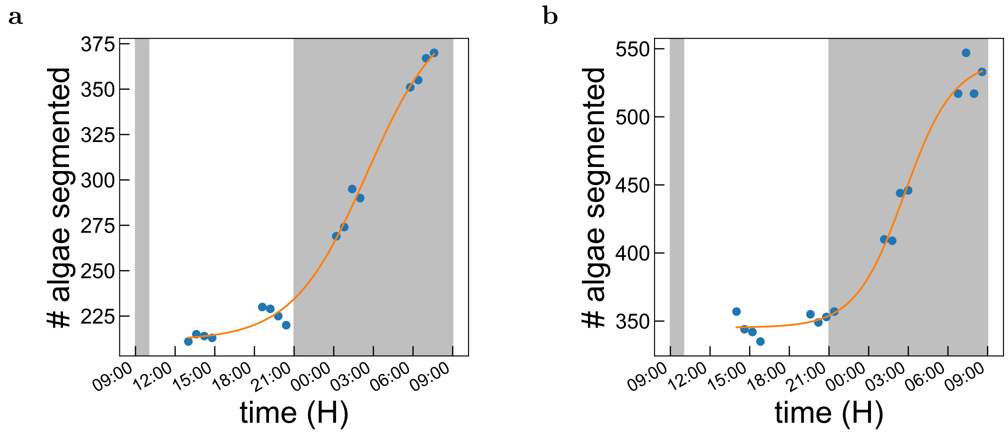

Figure S7: Evaluation of the synchronicity within *Chlamydomonas reinhardtii* population. Here the algae populations are the WT samples HL-treated under the microscope for the experiment described in Figure S4 of the Main Text. The algae are HL-treated under the microscope and taken out of the synchronization conditions, but the light-dark cycle to which they have been adapted is represented as dark areas in the figure. After a plateau during the expected light period, the algae start dividing at the onset of the expected dark period. The experiment is too short to assess that a plateau is reached after the division, but since the algae have been one day through HL-treatments and not in synchronized conditions, we can expect the synchronization to be lost after one day. **a,b**: number of algae segmented in the ChlF movie in the population *wt4a*$^-$ (resp. *cc*124) exposed to the light protocol under the microscope (see Main Text for sample conditions). A sigmoidal fit (orange line) performed on the data points gives a characteristic time of 2h50 (resp. 1h50).

because of the dark areas. A simplified way to find the center of a disk with mathematical morphology operation is the following. Using a binary mask of the object, multiple consecutive erosions of one pixel are performed. After each erosion, a dilation is used to test if the center of the object has been reached. Since the disk is convex, an erosion followed by a dilation is fully reversible, except in one case. If the pixel is not reconstructed by the dilation, it means that the sphere has been completely eroded, and that the pixel was the furthest from the sphere boundaries, ie. the disk center. The number of steps it took to reach this non-return step is the distance of the center to the edge, here the radius of the algae in pixels. The results are displayed in Figure S8.

We manually annotated a single image in order to evaluate the performance of the segmentation. We notice that the algae out of focus are not identified by the segmentation, which is not a problem because in practice they would receive a different light intensity and emit a low fluorescence signal resulting in a poor signal-to-noise ratio. It resulted in an F1-score of

73%.[3]

For long-lasting experiments as presented in Figure S11, the autofocus allows to keep the algae in the focal plane, but they can undergo horizontal shifts. It results in different positions of the algae between two videos. The algae are restored to their original position during the data processing by recovering the displacement vector between the two images. We recover it using a registration method based on the images cross-correlation [13].

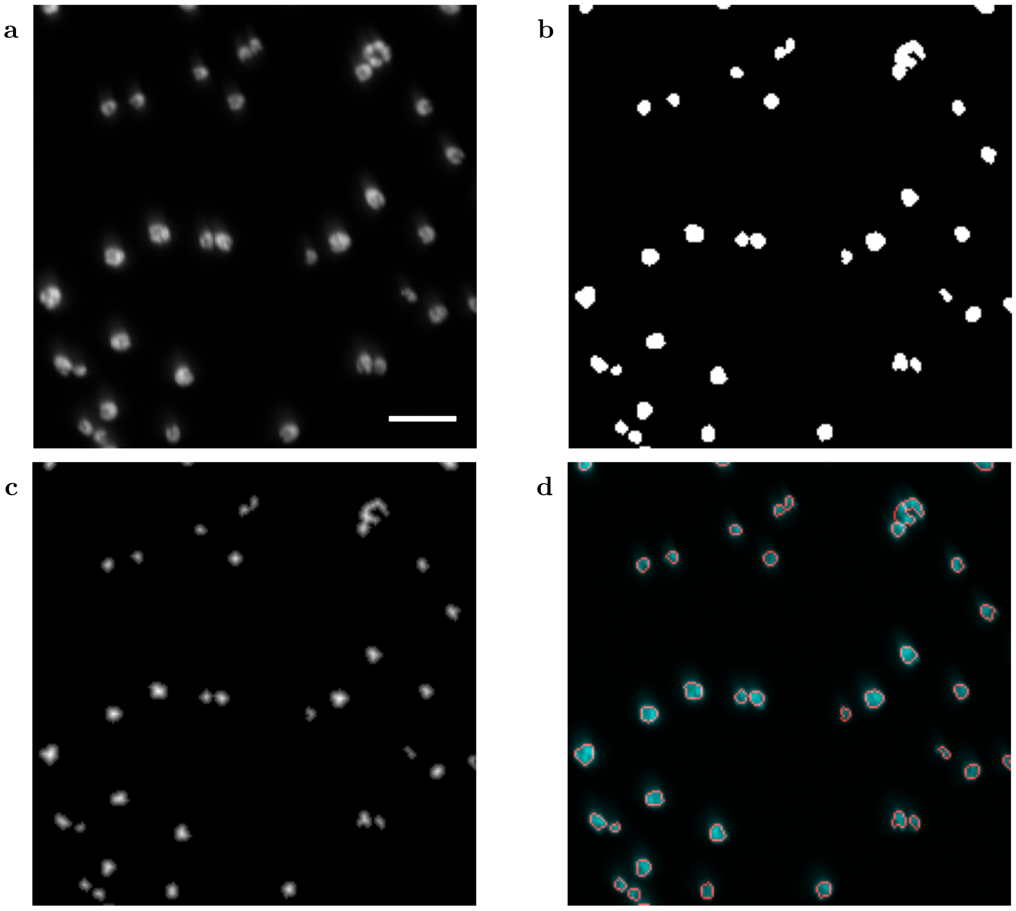

Figure S8: Watershed segmentation of the fluorescence movies. **a**: Grey-level image of the algae from a movie. Strain: *wt4a*$^{-}$. Scale: 30 µm, cropped image; **b**: Intersection of the contrast mask and the auto-level mask, used as reference for the watershed segmentation; **c**: Distance map used to identify the local maxima; **d**: Contours of the segmented objects.

2.6 Analysis of polypeptides

Total cellular proteins were resuspended in 200 mM dithiothreitol (DTT) and 200 mM Na_2CO_3 and solubilized in the presence of 2% SDS at 100°C for 60 s. Cell extracts were loaded on equal chlorophyll basis, separated by SDS-PAGE with 8 M urea [15], and electrotransferred onto nitrocellulose membranes (Amersham Protran 0.1 μm NC) by semi-dry method. Immunodetection was performed using antibodies against LHCSR [16], STT7 [17] and AtpB (beta-ATP synthase subunit) [18]. Signals were visualized by enhanced chemiluminescence (ECL - Clarity Biorad) and detected with a luminescent image analyzer ChemidocTM (Biorad).

[3] To compute the F1-score for segmentation, we first calculate the true positives (TP, pixels segmented as algae in the ground truth and predicted mask), false positives (FP - pixels segmented as algae that belong to background), and false negatives (FN - pixels segmented as background that are algae in the ground truth). These values can be derived from the overlapping regions between the predicted and ground truth masks. The precision (P) is defined as the ratio of true positives to the sum of true positives and false positives: precision = TP / (TP + FP). The recall (R) is defined as the ratio of true positives to the sum of true positives and false negatives: recall = TP / (TP + FN). The F1 score is then calculated as the harmonic mean of precision and recall: $F1 - score = 2\frac{P \times R}{P+R}$.

3 Data collection

3.1 Illumination protocols

3.1.1 Protocol inducing high-light stress and used to collect F_m' (reference protocol)

The reference protocol allows to induce simultaneously qE, qI and qT by applying a high light (400 μmol(photons) $\cdot$ m^{-2} $\cdot$ s^{-1}, 470$\pm$10 nm) for 15 min and turning it off. This light is 10 times above the growth light (50 μmol(photons) $\cdot$ m^{-2} $\cdot$ s^{-1}, white LED) corresponding to levels commonly used in the literature to induce light-stress response [19].

Simultaneously to the stressing light, we apply a saturating pulse every 20 s (1400 μmol(photons) $\cdot$ m^{-2} $\cdot$ s^{-1}, 405$\pm$7 nm, 200 ms). The blue light is turned off when the saturating pulse is applied, this allows to remove the fluorescence contribution in response to the blue light. It allows to compare the fluorescence response to a saturating pulse during the light stress, but also in the dark when the blue light is turned off. Figure S9 shows the time evolution of the light level in the field of view.

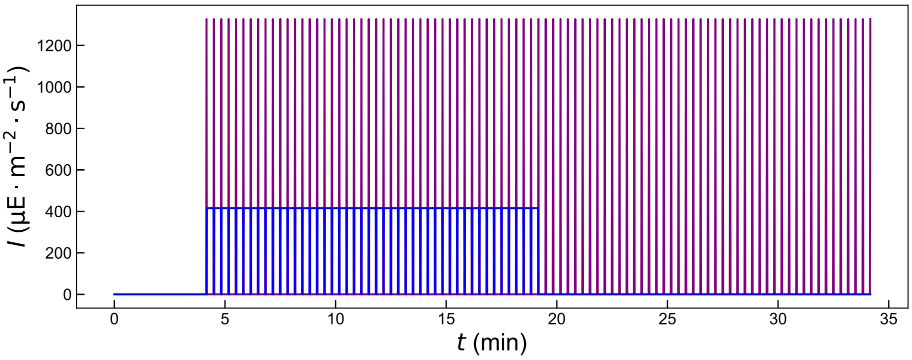

Figure S9: Description of the reference protocol illumination (I). The blue light induces the high-light stress (400 μmol(photons) $\cdot$ m^{-2} $\cdot$ s^{-1}, 470$\pm$10 nm), the violet light pulses (1400 μmol(photons) $\cdot$ m^{-2} $\cdot$ s^{-1}, 405$\pm$7 nm, 200 ms) saturate the photosynthetic chain to probe the fluorescence level under high-light and in the dark.

3.1.2 Summary of the light protocols

To complement the description in the methods section, the light protocols are summarized in Table S2. The typical excitation sequence of the protocol allowing to generate the training set is displayed in Figure S11a.

Name	Duration	Actinic light levels (μmol(photons) $\cdot$ m^{-2} $\cdot$ s^{-1})	**SP**	Figure
dark adaptation	15 min	0	OFF	S11
reference protocol	30 min	400 (470 nm)	ON	S9
four reference protocol repeats	2h	400 (470 nm)	ON	S11
HL-treatment	1h20-4h	400 (470 nm)	OFF	S11
low-light relaxation	45	40 (470 nm)	OFF	S11

Table S2: Summary of the light protocols. The actinic light is a LED (470$\pm$10 nm), the saturating pulses are produced with a LED (1400 μmol(photons) $\cdot$ m^{-2} $\cdot$ s^{-1}, 405$\pm$7 nm, 200 ms) applied every 20 s.

3.2 Evaluation of the quenching effect of the saturating pulses

In order to probe the fluorescence NPQ response under high-light and in the dark, we used a high saturating pulse frequency (1400 μmol(photons) $\cdot$ m^{-2} $\cdot$ s^{-1}, 405±7 nm, 200 ms) to have a better temporal resolution of the stress-responses, at the risk of provoking a stress related to the saturating pulse. Although actinic light already saturates and provokes stress, we assessed if the saturating pulses significantly affected the fluorescence response by applying the reference protocol with actinic blue light set to zero (Figure S10b) as opposed to the reference protocol illustrated in Figure S10a. Cells of *stt7-1* taken from growth light and transferred to MIN medium as indicated in Methods were exposed to this SP assessment sequence after the 15 min dark adaptation followed by the four reference protocol repeats. The time evolution of the fluorescence NPQ response to the SP assessment pattern is plotted in Figure S10d, whereas the response to the last repeat of the reference protocol that was applied previously is plotted in Figure S10c. The condition of this population corresponds to ***Pop_0*** and is expected to present minimal NPQ response. The stress-response in Figure S10c is still much richer with blue light on, demonstrating that the kinetics of ChlF response we observe mainly come from the light-stress provoked by the blue light, and not from the light-stress provoked by the saturating pulses. The slight increase of the response in Figure S10d may reflect the relaxation of the slowly-induced light stress response that we attribute to qI.

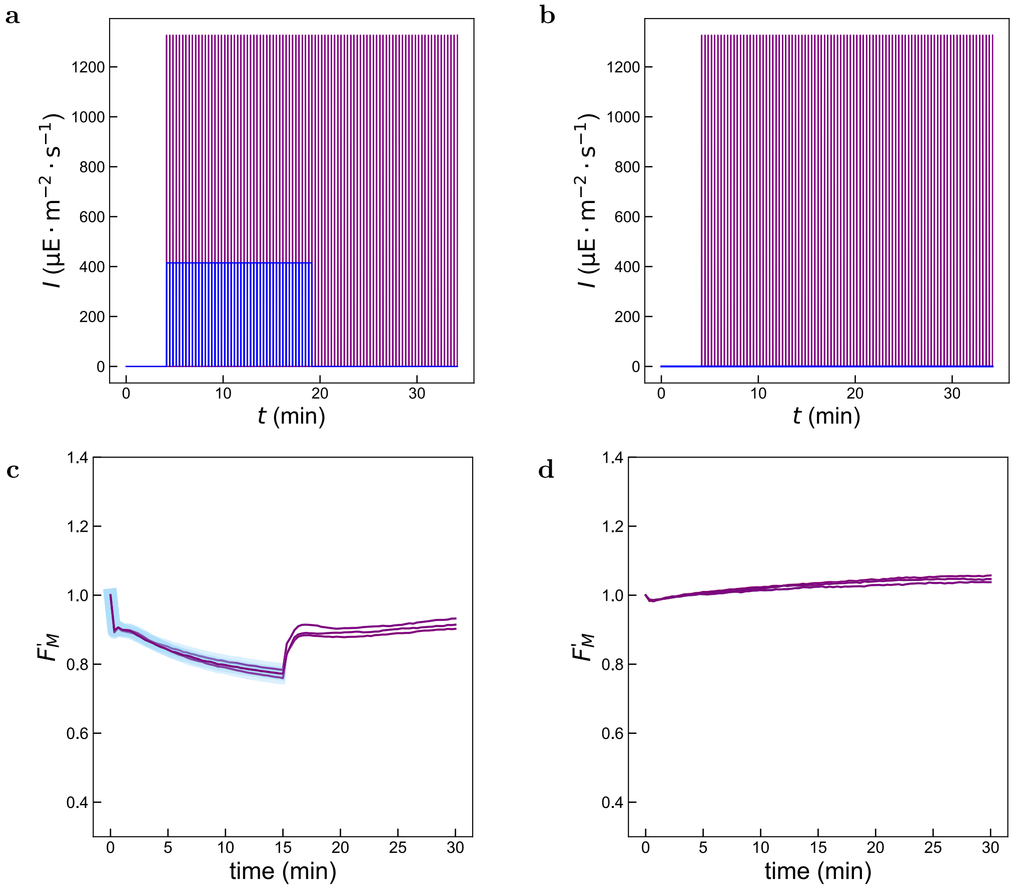

Figure S10: Evaluation of the quenching effect of the saturating pulse. **a**: Excitation sequence to investigate the light-stress induced by the saturating pulse when the blue light is turned on (reference protocol). **b**: Excitation sequence to investigate the light-stress induced by the saturating pulse when the blue light is turned off (SP assessment). **c**: Maximal fluorescence yield F_m' response of a *stt7-1* population to the last repeat of the four reference protocol repeats, before HL-treatment. Equivalent to ***Pop_0***. **d**: F_m' response of a *stt7-1* population to a SP assessment after 15 min dark adaptation and four reference protocol repeats.

3.3 Generation of the F_m' traces

The reference dataset was generated by applying the full protocol described in S11a to the populations *stt7-1* and *wt4a$^-$*. The summary of these conditions is provided in Table 1 of the Main text. Figure S11a shows the evolution of the blue light intensity received by the algae through the different steps, without the saturating pulses. There are no saturating

pulses during the HL-treatment and the relaxation, only during the reference protocol. Figure S11b,c show the fluorescence response to the consecutive light protocols for the two strains investigated (*wt4a*$^-$ and *stt7-1*). The reference protocol fluorescence response used to build the reference dataset as described in the Main Text are highlighted in the corresponding colors.

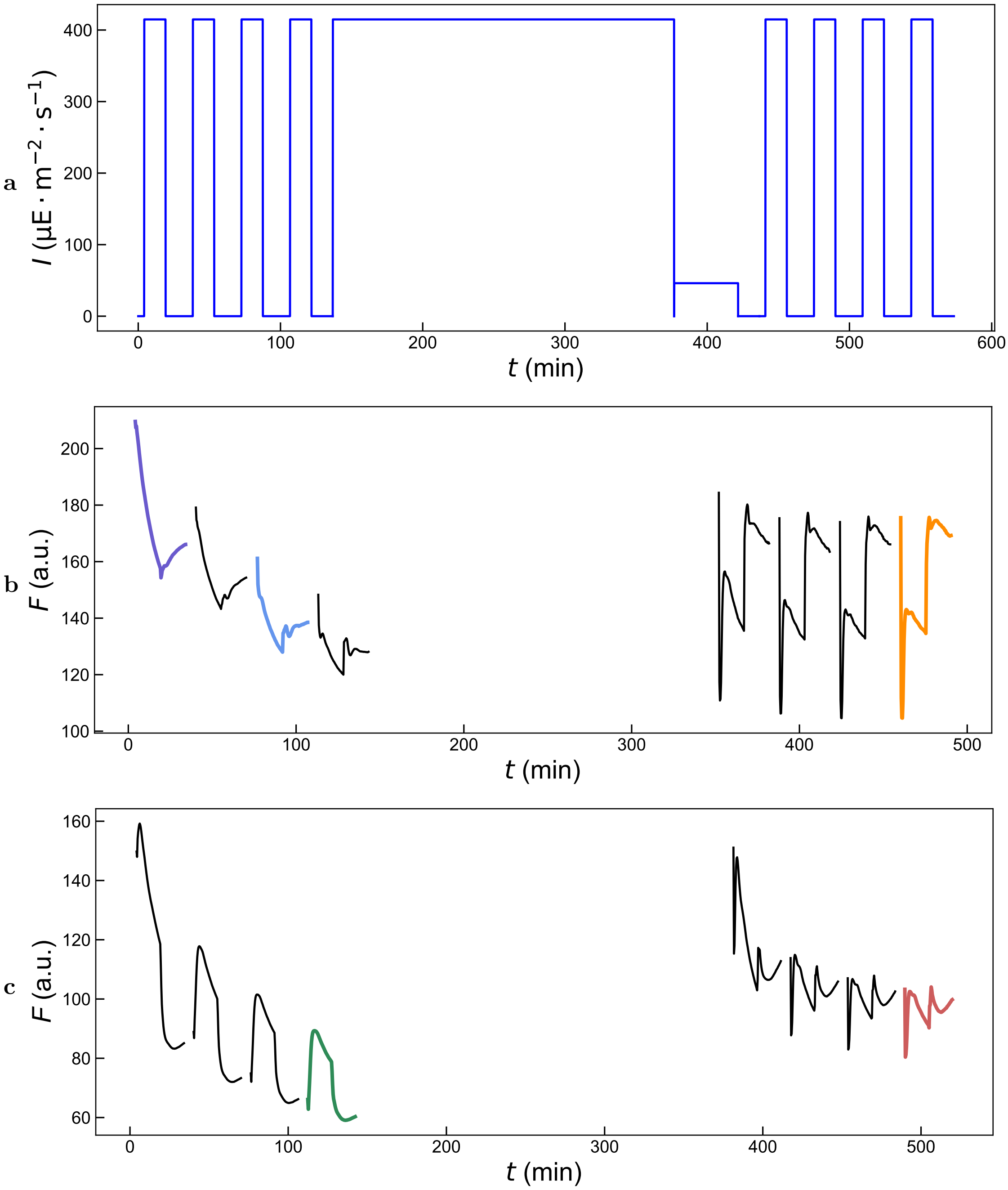

Figure S11: Full excitation sequence allowing to probe qE, qI, qT, activate qE and achieve a negligible contribution of qI. a: Intensity (I) of blue light inducing the high-light stress (saturating violet light pulses not displayed), the HL-treatment and the photoinhibition; **b,c**: Fluorescence (F) response of *stt7-1* (resp. *wt4a*$^-$) to the full protocol, average over the fluorescence of the algae in the videos. The timescale does not correspond exactly to the theoretical pattern because the video processing is integrated in the experiment and one experiment needs to terminate before another starts. The colors correspond the the reference protocols response selected to build the dataset. Violet: ***Pop_qI***, blue: ***Pop_0***, orange: ***Pop_qE***, green: ***Pop_qT***, red: *wt4a*$^-$.

The reference protocol was repeated four times in a row before and after HL-treatment to reduce the contribution of qI. We see that after the third repeat, the final F'_m is close to F_m for *stt7-1* after HL-treatment, and for *wt4a*$^-$ before and after HL-treatment. We selected these three types of responses to collect the F'_m traces of the populations ***Pop_qE***, ***Pop_qT*** and *wt4a*$^-$ respectively. For *stt7-1* before HL-treatment, we used the response to the first pattern to collect the F'_m traces of the population ***Pop_qI***. We notice that the exposure to

the reference protocol induces rapidly qE, as seen by the decrease of fluorescence response between two first pulses at the onset of the reference protocol. In order to minimize the effect of qE in this population of the dataset which is expected to show no stress response (***Pop_0***), we selected the third and not the fourth repeat of the reference protocol.

We also acquired similar data with monoclonal populations and *cc*124 populations that were not included in the dataset but used for investigation of the origin of the distribution of the NPQ scores (see Main Text).

For data processing, the F'_m traces, response to saturating pulses only, were isolated from the whole fluorescence trace by selecting one in every 20 frames (framerate: 1 Hz, saturating pulse applied every 20 s). The data normalization is discussed in Section 4.

The dataset as well as a description of how to use it are available online [4]

[4]https://github.com/DreamRepo/NPQScore-data

4 Dictionary learning to represent the F'_m traces

4.1 Investigation of the hyperparameter space

We explored several parameters, based on the analysis of the reconstruction error.

- **Normalization method**: We tried normalizing F'_m by
  - the standard deviation $\sigma = \sqrt{\frac{1}{N-1}\sum_{i=1}^{N}(F'_m(i) - \bar{F'_m})^2}$, a classical reduction method;
  - the first pulse value $F'_m(0)$ (also referred to as F_m);
  - the sum S $= \sum_{i=1}^{N} F'_m(i)$ to obtain a probability law.
- **Dictionary Size** N_D: The size of the dictionary determines the number of atoms to be learned. The higher the size, the better the reconstruction. We explored sizes of dictionaries from 3 to 30 atoms.
- **Sparsity Level** λ: The sparsity enforces to learn to reconstruct the traces by combining a minimal number of atoms of the Dictionary. We explored sparsity constraints between 10^{-10} and 10^{-2}.
- **Training set size** $n_{samples}$: This parameter corresponds to the number of F'_m traces representing each class in the training. We used a balanced dataset, by selecting $n_{samples}$ in each class. The total training size is $4 \times n_{samples}$ because we have four classes. We tested values of $n_{samples}$ between 3 to 300 (representative of the size of the smallest class in the reference dataset).

Figure S12a and b show a poor and good reconstruction of a traces from HL-treated $wt4a^{-}$ population with a dictionary of 2 and 10 atoms respectively. Several number of training-set sizes, number of atoms and sparsity levels were explored to see their influence on the reconstruction error. We began by a grid search, splitting the exploration space by choosing seven values each for N_D, λ and $n_{samples}$. Then, we refined the size of the search space around the optimum.

We selected the normalization by S after observing that it systematically yielded the lowest reconstruction error compared to the other normalization methods (Figure S12c). In the same Figure, we identify that the reconstruction error decreases with the number of atoms in the dictionary. In practice, when using the normalization by the sum S, and a dictionary larger than 12 atoms, some atoms were never used in the reconstruction of the training set, and were therefore irrelevant to train the LDA. This was true for all the values of λ we investigated (10^{-10} to 10^{-2}). It is why the curve of the normalization by S stops at 12. The Figures S12d and S12e correspond to the results of the investigation limited to the normalization by S for clarity.

We investigated the impact of the size of the training set on the reconstruction error. Figure S12d shows that it is much less significant than the number of atoms. It shows that even a low amount of training data is sufficient to encompass the dynamics of the NPQ components in the dictionary. Therefore, the method could be generalized to studies at the population level with only a few repeats. Based on the curves, we selected 10 atoms for our dictionary, with a corresponding reconstruction error threshold of $2{\cdot}10^{-4}$.

Finally, we investigated the role of the parameter λ on the reconstruction, as shown in Figure S12e (normalization by S, number of atoms: 10). Given the span of the error axis, we can deduce that it is minimal. It does however play a role on the number of atoms used for the reconstruction as we observed experimentally. We selected $\lambda = 10^{-6}$ to give maximal weight to the sparsity constraint in the metric optimization.

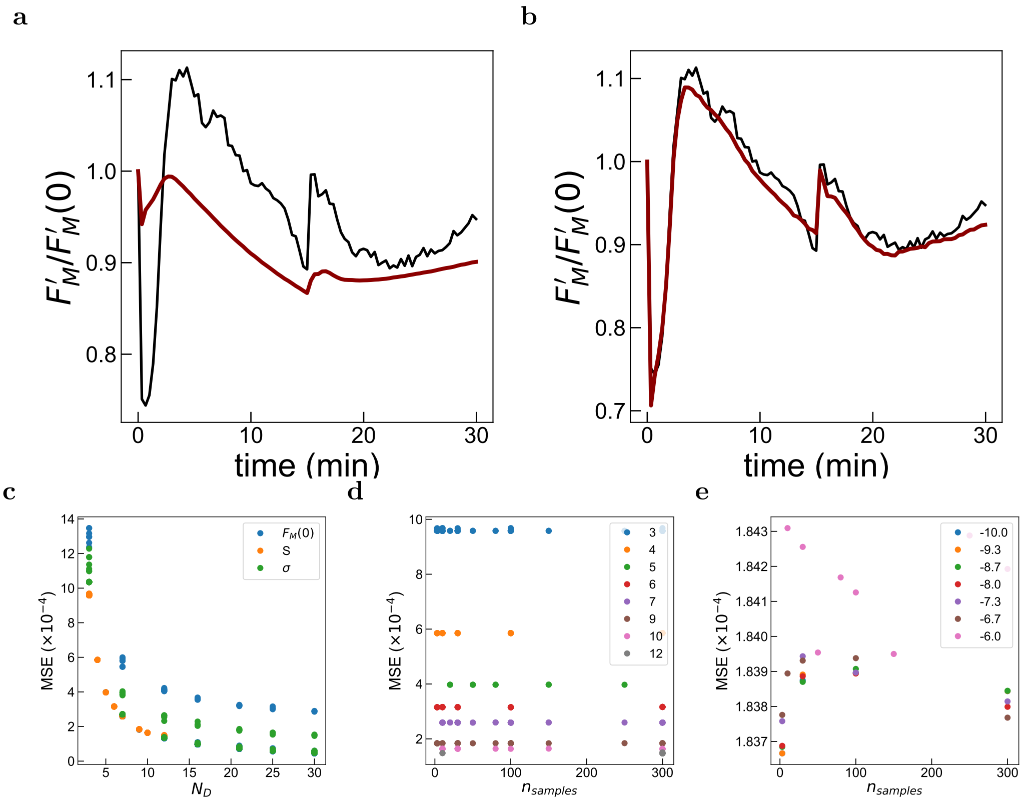

Figure S12: Exploration of the role of the hyperparameters of the Dictionary Learning on the reconstruction error. **a**: Fluorescence trace (black, *wt4a*$^-$ HL-treated) and trace reconstructed with the dictionary learning method (red), with $N_D = 2$, using the normalization by $F'_m(0)$ and $n_{samples} = 300$. The mean squared error (MSE) equals $6.7{\times}10^{-3}$ and $r^2 = -0.13$. **b**: Fluorescence trace (black) and trace reconstructed with the dictionary learning method (red), with $N_D = 10$, using the normalization by S and $n_{samples} = 300$. The mean squared error equals $2.6{\times}10^{-4}$ and $r^2 = 0.95$. **c**: Impact of the normalization method and the number of atoms N_D on the reconstruction error. Legend: normalization method; **d**: Impact of the training set size $n_{samples}$ and the number of atoms N_D for dictionaries built using the normalization by S. Legend: number of samples per population (balanced dataset). **e**: Impact of the training set size $n_{samples}$ and the sparsity level λ for dictionaries built using the normalization by S and 10 atoms. Legend: $\log(\lambda)$.

4.2 Validation of the reconstruction on F'_m traces

We evaluated the performances of the first dimension reduction step (dictionary learning method) at reconstructing new data. First we investigated its capacity at reconstructing a validation set: monoclonal populations equivalent to ***Pop_0***, ***Pop_qE***, ***Pop_qT*** and ***Pop_qI*** not present in the training set. In figure S13b-e, we show traces overlayed with the dictionary reconstruction (seven traces with the poorest reconstruction from each class). We evaluate the quality of the reconstruction by computing the mean-squared error (MSE) and plot the MSE for each class in Figure S13j. We can identify a maximal average reconstruction error for the class ***Pop_qE*** at MSE = 1.10^4. In Figure S13f-i, we plot the reconstruction error for experimental datasets that were absent from the training set, at the fourth repeat, before and after HL-treatment. They consist of two wild-type populations, wild type *wt4a*$^-$ and wild type *cc124*. All these strains are expected to display qT, and qE when they are HL-treated. In all cases, the MSE is contained within the range of error of the training set (Figure S13k), therefore we can conclude that the dictionary reconstruction generalizes well to new data that combine the training traits. It serves as validation of the initial hypothesis that a fluorescence trace of combined traits is well represented as a linear combination of the fluorescence traces corresponding to individual traits.

5 Linear Discriminant Analysis (LDA) to project the dataset onto the NPQ space

We did not quantitatively analyze the slower NPQ component in the Main Text since this biological phenomenon is the hardest to control among the ones investigated and since no mutant strain exists. Accordingly, once we obtained the machine learning framework, we

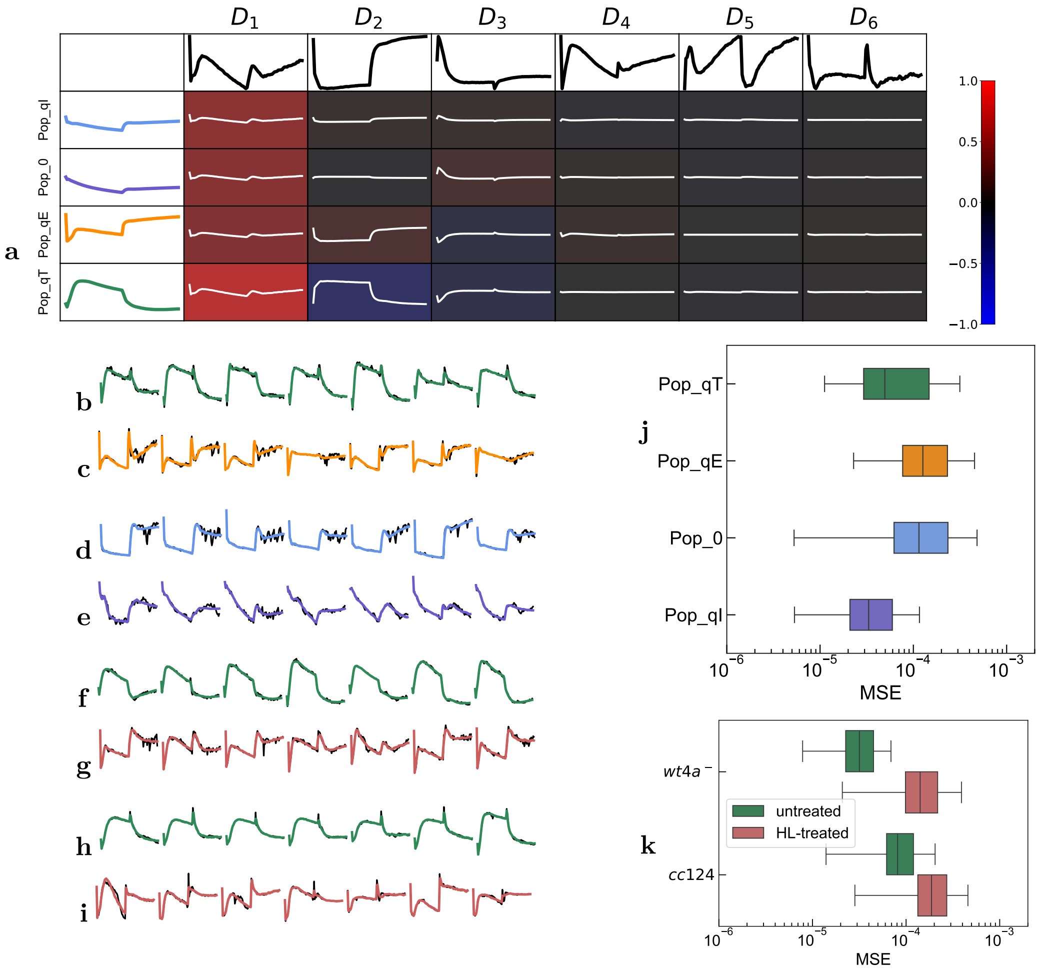

Figure S13: Dictionary learning reconstruction for phenotypes absent from the training set. **a**: The atoms D_i are the learned representation of the dataset. Each element of the dataset can be reconstructed as a linear combination of the atoms D_i. In the table, the first column shows the reconstruction of the average trace of each population in the reference dataset. In each table cell, the color scales Z_i while the trace is $Z_i \times D_i$; **b-e**: Raw (black) and reconstructed (colors) traces of the monoclonal populations not seen by the training set with the dictionary. The seven reconstructions with the highest Mean-Squared Error (MSE) are displayed. **b**: ***Pop_qT***, **c**: ***Pop_qE***, **d**: ***Pop_0***, **e**: ***Pop_qI***; **f-i**: Raw (black) and reconstructed ChlF traces of populations absent from the training set (except ***Pop_qT*** in **f**). The ChlF traces are measured before (green) and after (red) HL-treatment for 2-4 h. **f,g**: *wt4a*$^-$, **h,i**: **cc124** ; **j**: Distribution of the reconstruction mean squared error from monoclonal experiments not seen by the training set. ***Pop_qT***: 180 algae, ***Pop_qE***: 380 algae, ***Pop_0***: 317 algae, ***Pop_qI***: 326 algae; **k**: Distribution of the reconstruction mean squared error from experiments displayed in **f-i** *wt4a*$^-$: 196 algae, *cc*124: 164 algae. The traces plotted in **b-i** have the highest reconstruction error among all the algae in the experiment.

focused on populations where qI could be neglected.

Figure S14 visually demonstrates that the amplitude of characteristic feature for the qI, ie. global decrease of the fluorescence throughout the whole experiment with a final fluorescence level F'_m not recovering to the initial value, increases as the corresponding datapoint moves away from the origin. Notably, on the q̃I axis, the distance of ***Pop_qI*** from the origin is only slightly larger than that of the other training populations, indicating that this NPQ component is smaller and not as tightly controlled as the other two in the reference dataset. This might reflect the photoinhibited state of *stt7-1* even under low-light growth conditions .

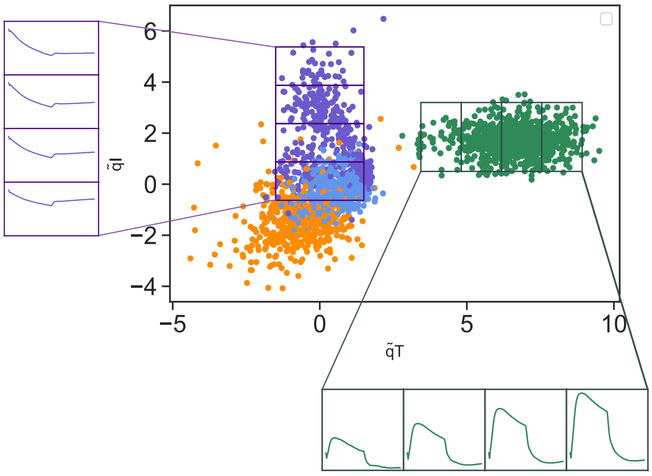

Figure S14: Qualitative analysis of the evolution of the fluorescence traces along the q̃I and q̃T axis. The violet population corresponds to the population ***Pop_qI*** (*stt7-1* before HL-treatment, first repeat). The span of (98% of) the population along the q̃I axis is split into 4 equal parts, and the average fluorescence trace within each quarters are computed using the fluorescence traces (normalized to the first point) corresponding to the data points falling inside the quarter. The same is performed for the ***Pop_qT*** population (green point cloud, $wt4a^{-}$ before HL-treatment, fourth repeat) along the q̃T axis. For all plots, the span of the y-axis is equal to 0.7, with the first point falling at position 1.

6 Comparison with *ad hoc* metrics

To validate the method, we used *ad hoc* features identified in the literature allowing to derive quantitative values to represent the stresses. We compare the so-called relative NPQ values to the axis values and evaluated their correlation.

To evaluate the NPQ of a F'_m trace of saturating pulses F'_m, the formula described in the literature is $NPQ(t_n) = \frac{F_m(0)-F_m(t_n)}{F_m(t_n)}$. We compare the NPQ($t_n$) values against the metrics we extracted with the machine learning pipeline in order to see if our findings are consistent with the literature. The fluorescence value at t_n is highlighted on the top of the graphs, over the average traces of the training set overlaid. Several characteristic phases of the F'_m traces have been identified to reflect either qE, qT and qI, based on mutants experiments [20–22]. We attributed time values of interest t_n based on the local extrema described in [20] to identify them in each experiment. As described in the main text:

- The sharp decay of fluorescence at the light onset has been attributed to qE [20] and corresponds to NPQ(t_1) (first SP after 10 s of HL). We compare it to the values computed for q̃E for all the algae in the class ***Pop__qE*** of the dataset and display the results in figure S15a.

- The gradual increase of the fluorescence under the light has been attributed to the migration of light-harvesting antennas to PSII and corresponds to NPQ(t_{45}) (last SP after 15 min of HL). We compare it to the values computed for q̃T for all the algae in the class ***Pop__qT*** of the dataset and display the results in Figure S15b.

- The effect of qI contributes to a non-reversible decay of the fluorescence throughout the whole experiment (NPQ(t_{91}) - last SP after 15 min in the dark following the 15 min of HL). We compare it to the values computed for q̃I for all the algae in the class ***Pop__qI*** of the dataset and display the results in Figure S15c.

In all cases we find a high correlation between the two scores for the class of interest.

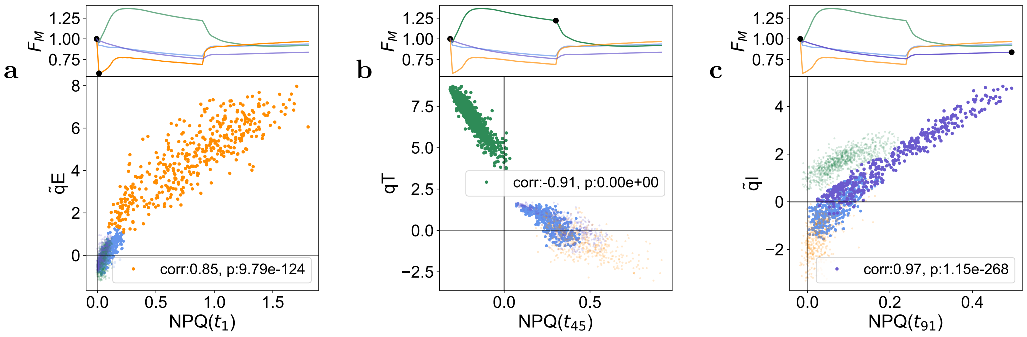

Figure S15: Comparison of the non photochemical quenching (NPQ) features identified in the literature with the metrics extracted with the machine learning pipeline. The average fluorescence traces of the training set are displayed on top, while the NPQ metrics are plotted against the scores computed with the machine learning pipeline. **a** shows the comparison of the effect of qE, contributing to the non-reversible decay of the fluorescence throughout the whole experiment (NPQ(t_1)), with the values computed for q̃E for all the algae in the training set. The correlation coefficient displayed corresponds to the class ***Pop__qE*** (orange, *stt7-1* after HL-treatment). **b** shows the comparison of the effect of qT, contributing to the non-reversible decay of the fluorescence throughout the whole experiment (NPQ(t_{45})), with the values computed for q̃T for all the algae in the training set. The correlation coefficient displayed corresponds to the class ***Pop__qT*** (green, *wt4a*$^-$ before HL-treatment). **c** shows the comparison of the effect of qI, contributing to the non-reversible decay of the fluorescence throughout the whole experiment (NPQ(t_{91})), with the values computed for q̃I for all the algae in the training set. The correlation coefficient displayed corresponds to the class ***Pop__qI*** (violet, *stt7-1* before HL-treatment, first repeat).

7 Statistical tests

7.1 Monoclonal experiments

In order to evaluate the reproducibility of the NPQ score, we performed a Mann-Whitney test on each couple of scores from two consecutive repeats on the monoclonal populations used in the experiments presented in Figure S3 of the Main Text. As displayed in Figure S16, the hypothesis of equality of the underlying distributions of the data was rejected for the $\tilde{q}$I scores of ***Pop_qT*** (p-value 10^{-6}) and $wt4a^{-}$ (p-value 10^{-3}). For all the other samples, we failed to reject the equality hypothesis (p-values > 0.04). Therefore, we could use these experiments to study $\tilde{q}$E and $\tilde{q}$T by identifying the repeats as identical.

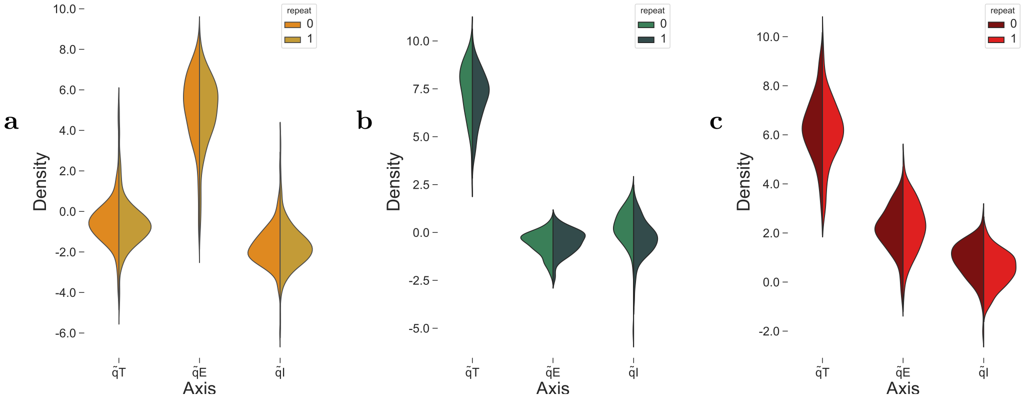

Figure S16: Distribution of the non-photochemical quenching (NPQ) scores for the two consecutive repeats (third and fourth) of the experiments on monoclonal populations. **a**: ***Pop_qE*** (*stt7-1* after HL-treatment) monoclonal, two repeats – failed to reject equality ; **b**: ***Pop_qT*** ($wt4a^{-}$ before HL-treatment) monoclonal, two repeats – failed to reject equality except for $\tilde{q}$I (p-value 10^{-6}); **c**: $wt4a^{-}$ HL-treated, monoclonal, two repeats – failed to reject equality except for $\tilde{q}$I (p-value 10^{-3}). Violin width represent data density.

7.2 Main text figures

The various statistical tests performed on the elements presented in the Main Text are presented in Table S3. The null-hypothesis is described along with the p-values and the conclusion. We performed normality tests on the data and report on the sample sizes. The data compared often share the same size, except when comparing $D_{i,i}$ and $D_{i,j}$. In the latter case, we used a bootstraping method, by performing the test on subsets of $D_{i,j}$ with sizes equal to $D_{i,i}$ (100 draws). The tests were performed in Python with the SciPy statistics library functions [23].

Figure	Population	Variable	Size	Null hypothesis	Samples compared	Test	p-value	Conclusion
3B	***Pop_qE*** monoclonal	$D_{i,i}$	176	the central tendancy of the distribution of X is greater then Y's.	X = $D_{i,i}$ and Y = $D_{i,j}$	Mann-Whitney bootstrap $D_{i,j}$	10^{-33}	rejected
		$D_{i,j}$	30976					
3E	***Pop_qT*** monoclonal	$D_{i,i}$	77	the central tendancy of the distribution of X is greater then Y's.	X = $D_{i,i}$ and Y = $D_{i,j}$	Mann-Whitney bootstrap $D_{i,j}$	10^{-9}	rejected
		$D_{i,j}$	5929					
3A	repeat 0	q̃E	176	the underlying distributions of q̃E in the two repeats are equal	two consecutive repeats	Mann-Whitney	0.25	failed to reject
	repeat 1	q̃E	176					
3E	repeat 0	q̃T	77	the underlying distributions of q̃T in the two repeats are equal	two consecutive repeats	Mann-Whitney	0.04	failed to reject
	repeat 1	q̃T	77					
3C	***Pop_qE***	q̃E		all input samples are from populations with equal variances	3 non-monoclonal<br>2 monoclonal	Levene	0.99	failed to reject
3F	***Pop_qT***	q̃T		all input samples are from populations with equal variances	4 non-monoclonal<br>1 monoclonal	Levene	10^{-9}	rejected[1]
3I	*wt4a*$^-$ monoclonal	$D_{i,i}$	77	the central tendancy of the distribution of X is greater then Y's.	X = $D_{i,i}$ and Y = $D_{i,j}$	Mann-Whitney bootstrap $D_{i,j}$	10^{-6}	rejected
		$D_{i,j}$	5929					
3H	repeat 0	q̃T	77	the underlying distributions of q̃T in the two repeats are equal	two consecutive repeats	Mann-Whitney	0.4	failed to reject
	repeat 1	q̃T	77					
3G	repeat 0	q̃E	77	the underlying distributions of q̃E in the two repeats are equal	two consecutive repeats	Mann-Whitney	0.5	failed to reject
	repeat 1	q̃E	77					
4A	***Pop_qT*** to *wt4a*$^-$	q̃E, q̃T	200-800	the underlying distributions of q̃E and q̃T are uncorrelated	q̃E and q̃T for the total population	Pearson	$<10^{-250}$	rejected
		q̃E, q̃T	200-800		q̃E and q̃T for individual sequentially activated populations		$<10^{-37}$	rejected
4D	cc124 to *cc124	q̃E, q̃T	300-800	the underlying distributions of q̃E and q̃T are uncorrelated	q̃E and q̃T for the total population	Pearson	$<10^{-230}$	rejected
		q̃E, q̃T	300-800		q̃E and q̃T for individual sequentially activated populations		<0.3	failed to reject[2]

Table S3: Statistical tests performed on the elements presented in the Main Text. [1] the test was rejected due to the diversity of variance in the non-monoclonal strains, but the monoclonal variance is not an outlier. [2] Accepted only once for 80 min of activation, with other values smaller than 10^{-40}. $D_{i,i}$: cell-to-cell distance in 3D NPQ space, $D_{i,j}$: cell-to-puppluation distance in 3D NPQ space

8 Sources of heterogeneity

In order to test the correlation between the heterogeneity of illumination of the algae and the level of expression of qE, qT, and qI , we first exploited the calibration of the light intensity in the field of view with **Dronpa-2** (Figure S3). We then measured the NPQ-score of the monoclonal culture wild type *wt4a*$^-$ HL-activated over four reference protocol repeats. As displayed in Figure S17a–c, no significant correlation appears.

In order to test the contribution of the size of the algae to the variance of the NPQ scores, we also extracted the projected surface of each alga exposed to the light as derived from the number of pixels corresponding to the segmented alga. As shown in Figure S17d–e, we did not evidence any significant correlation.

Eventually, we evaluated if the spatial position of the algae could affect their NPQ score. Indeed, they may have been differently exposed to gas exchange from the sides of the observation chamber. As displayed in Figure S17g–i, we did not evidence any specific spatial pattern of the NPQ score.

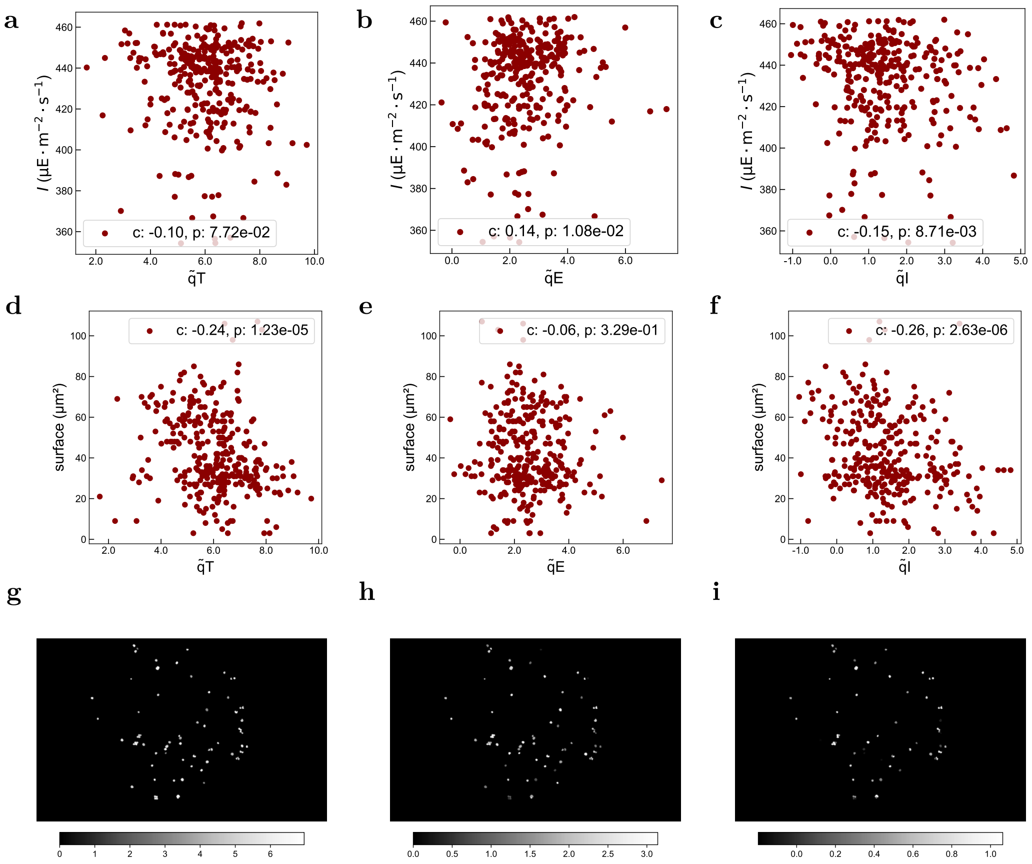

Figure S17: Investigation of the correlation of the non-photochemical quenching (NPQ) score with various parameters. a–**c**: Dependence of the NPQ score (**a**: $\tilde{q}$T; **b**: $\tilde{q}$E; **c**: $\tilde{q}$I) on light intensity (I); **d**–**f**: Dependence of the NPQ score (**d**: $\tilde{q}$T; **e**: $\tilde{q}$E; **f**: $\tilde{q}$I) on the cell size; **g**–**i**: Dependence of the NPQ score (**g**: $\tilde{q}$T; **h**: $\tilde{q}$E; **i**: $\tilde{q}$I) on the cell position in the observation chamber. Strain: monoclonal *wt4a*$^-$ HL-activated, **a-f** show overlap of the four consecutive reference protocol responses.

9 Evaluation of the relevance of the axis q̃I

To ensure the single-cell level correlation between q̃E and q̃T observed in wild type $wt4a^{-}$ (Figure 4A in the Main Text) did not actually originate from a hidden effect from q̃I, we studied the correlation between q̃E and q̃T in subpopulations of wild type $wt4a^{-}$ with different q̃I levels. We selected a low q̃I population (score q̃I<-2) and a high q̃I population (score q̃I>1). The scatter plot of q̃E and q̃T in these two subpopulations is displayed in Figure S18. We observe that the correlation between q̃E and q̃T remains strong in both subpopulations, indicating that the correlation observed in Figure 4A of the Main Text is not an artifact due to q̃I.

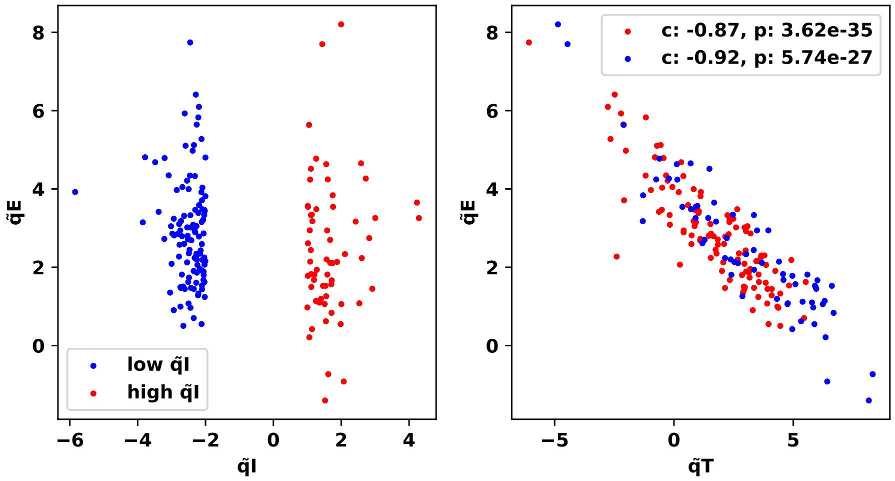

Figure S18: Correlation between q̃E and q̃T in subpopulations of wild type $wt4a^{-}$ **with low and high q̃I.**
Left: Scatter plot of the q̃E and q̃I scores in the subpopulation of wild type $wt4a^{-}$ corresponding to low and high q̃I levels. Right: Scatter plot of the q̃E and q̃T scores for low q̃I and high q̃I populations. The Pearson correlation coefficient is $c = -0.87$ (resp. $c = -0.92$) with a p-value 10^{-35} (resp. 10^{-27}) for the low (resp. high) q̃I population.

References

[1] J. Verhoeven. "Glossary of terms used in photochemistry (IUPAC Recommendations 1996)". In: *Pure and Applied Chemistry* 68.12 (1996), pp. 2223–2286.

[2] A. Lahlou et al. "Fluorescence to Measure Light Intensity". In: *Nature Methods* 20.12 (Dec. 2023), pp. 1930–1938. DOI: 10.1038/s41592-023-02063-y.

[3] R. Zhang et al. "Macroscale Fluorescence Imaging against Autofluorescence under Ambient Light". In: *Light: Science & Applications* 7.1 (Nov. 2018), p. 97. DOI: 10.1038/s41377-018-0098-6.

[4] J. Pech-Pacheco et al. "Diatom Autofocusing in Brightfield Microscopy: A Comparative Study". In: *Proceedings 15th International Conference on Pattern Recognition. ICPR-2000.* Vol. 3. Sept. 2000, 314–317 vol.3. DOI: 10.1109/ICPR.2000.903548.

[5] L. Girolomoni et al. "LHCSR3 Is a Nonphotochemical Quencher of Both Photosystems in Chlamydomonas Reinhardtii". In: *Proceedings of the National Academy of Sciences* 116.10 (Mar. 2019), pp. 4212–4217. DOI: 10.1073/pnas.1809812116.

[6] G. Peers et al. "An Ancient Light-Harvesting Protein Is Critical for the Regulation of Algal Photosynthesis". In: *Nature* 462.7272 (Nov. 2009), pp. 518–521. DOI: 10.1038/nature08587.

[7] K. Kosuge et al. "LHCSR1-dependent Fluorescence Quenching Is Mediated by Excitation Energy Transfer from LHCII to Photosystem I in Chlamydomonas Reinhardtii". In: *Proceedings of the National Academy of Sciences* 115.14 (Apr. 2018), pp. 3722–3727. DOI: 10.1073/pnas.1720574115.

[8] M. Cantrell, M. A. Ware, and G. Peers. "Characterizing Compensatory Mechanisms in the Absence of Photoprotective qE in Chlamydomonas Reinhardtii". In: *Photosynthesis Research* 158.1 (Oct. 2023), pp. 23–39. DOI: 10.1007/s11120-023-01037-7.

[9] M. Á. Ruiz-Sola et al. "Light-Independent Regulation of Algal Photoprotection by CO2 Availability". In: *Nature Communications* 14.1 (Apr. 2023), p. 1977. DOI: 10.1038/s41467-023-37800-6.

[10] E. Tyystjärvi and E.-M. Aro. "The Rate Constant of Photoinhibition, Measured in Lincomycin-Treated Leaves, Is Directly Proportional to Light Intensity". In: *Proceedings of the National Academy of Sciences of the United States of America* 93 (Apr. 1996), pp. 2213–8. DOI: 10.1073/pnas.93.5.2213.

[11] E. Tyystjärvi. "Chapter Seven - Photoinhibition of Photosystem II". In: *International Review of Cell and Molecular Biology.* Ed. by K. W. Jeon. Vol. 300. International Review of Cell and Molecular Biology. Academic Press, Jan. 2013, pp. 243–303. DOI: 10.1016/B978-0-12-405210-9.00007-2.

[12] M. Nilkens et al. "Identification of a Slowly Inducible Zeaxanthin-Dependent Component of Non-Photochemical Quenching of Chlorophyll Fluorescence Generated under Steady-State Conditions in Arabidopsis". In: *Biochimica et Biophysica Acta (BBA) - Bioenergetics* 1797.4 (Apr. 2010), pp. 466–475. DOI: 10.1016/j.bbabio.2010.01.001.

[13] R. Chouket et al. "Extra Kinetic Dimensions for Label Discrimination". In: *Nature communications* 13.1 (2022), pp. 1–8.

[14] Alienor134. *Alienor134/Image_segmentation:* Zenodo. Nov. 2021. DOI: 10.5281/zenodo.5684343.

[15] R. G. Piccioni, N.-H. Chua, and P. Bennoun. "A Nuclear Mutant of *Chlamydomonas reinhardtii* Defective in Photosynthetic Photophosphorylation: Characterization of the Algal Coupling Factor ATPase". en. In: *European Journal of Biochemistry* 117.1 (June 1981), pp. 93–102. DOI: 10.1111/j.1432-1033.1981.tb06307.x.

[16] G. Bonente et al. "Analysis of LhcSR3, a Protein Essential for Feedback De-Excitation in the Green Alga Chlamydomonas Reinhardtii". In: *PLOS Biology* 9.1 (Jan. 2011), e1000577. DOI: 10.1371/journal.pbio.1000577.

[17] S. V. Bergner et al. "STT7-Dependent Phosphorylation Is Modulated by Changing Environmental Conditions, and Its Absence Triggers Remodeling of Photosynthetic Protein Complexes". In: *Plant Physiology* 168.2 (June 2015), pp. 615–634. DOI: 10.1104/pp.15.00072.

[18] A. Atteia et al. "Identification of mitochondrial proteins in membrane preparations from Chlamydomonas reinhardtii." In: *Journal of Biological Chemistry* 267.1 (Jan. 1992), pp. 226–234. DOI: 10.1016/S0021-9258(18)48483-9.

[19] U. Schreiber. "Pulse-Amplitude-Modulation (PAM) Fluorometry and Saturation Pulse Method: An Overview". In: *Chlorophyll a Fluorescence: A Signature of Photosynthesis*. Ed. by G. C. Papageorgiou and Govindjee. Advances in Photosynthesis and Respiration. Dordrecht: Springer Netherlands, 2004, pp. 279–319. ISBN: 978-1-4020-3218-9. DOI: 10.1007/978-1-4020-3218-9_11.

[20] G. Allorent et al. "A Dual Strategy to Cope with High Light in Chlamydomonas Reinhardtii". In: *The Plant Cell* 25.2 (Feb. 2013), pp. 545–557. DOI: 10.1105/tpc.112.108274.

[21] M. Á. Ruiz-Sola and D. Petroutsos. "A Toolkit for the Characterization of the Photoprotective Capacity of Green Algae". In: *Plastids*. Ed. by E. Maréchal. Vol. 1829. New York, NY: Springer US, 2018, pp. 315–323. ISBN: 978-1-4939-8653-8 978-1-4939-8654-5. DOI: 10.1007/978-1-4939-8654-5_21.

[22] T. Roach and C. S. Na. "LHCSR3 Affects De-Coupling and Re-Coupling of LHCII to PSII during State Transitions in Chlamydomonas Reinhardtii". In: *Scientific Reports* 7.1 (Mar. 2017), p. 43145. DOI: 10.1038/srep43145.

[23] P. Virtanen et al. "SciPy 1.0: Fundamental Algorithms for Scientific Computing in Python". In: *Nature Methods* 17.3 (Mar. 2020), pp. 261–272. DOI: 10.1038/s41592-019-0686-2.
